# Non-pharmacological therapies of traditional Chinese medicine in prediabetes: a systematic review and network meta-analysis of randomized controlled trials

**DOI:** 10.3389/fendo.2026.1907358

**Published:** 2026-08-27

**Authors:** Ximing Qi, Tianzuo Wang, Lin Liao

**Affiliations:** 1 First Clinical Medical College, Shandong University of Traditional Chinese Medicine, Jinan, China; 2 Department of Endocrinology and Metabology, Shandong Provincial Qianfoshan Hospital, Shandong University of Traditional Chinese medicine, Jinan, China; 3 Department of Endocrinology and Metabology, The First Affiliated Hospital of Shandong First Medical University & Shandong Provincial Qianfoshan Hospital, Shandong Key Laboratory of Rheumatic Disease and Translational medicine, Shandong Institute of Nephrology, Jinan, China

**Keywords:** network meta-analysis, non-pharmacological therapy, prediabetes, systematic review, traditional Chinese medicine

## Abstract

**Background:**

Prediabetes is an independent risk factor for diabetes complications and all-cause mortality. Non-pharmacological therapies of traditional Chinese medicine (NPTTCM) offer promising alternatives for prediabetes, yet their comparative efficacy remains unclear.

**Objective:**

The present study aimed to evaluate the efficacies of 6 NPTTCM (acupuncture, acupoint catgut embedding (ACE), Baduanjin, electroacupuncture, massage and Taiji) in prediabetes intervention.

**Methods:**

Eight databases were searched for randomized controlled trials (RCTs). The network meta-analyses were performed to estimate mean differences and 95% confidence intervals and surface under the cumulative ranking curve (SUCRA) was used to rank NPTTCM.

**Results:**

Fifty-one RCTs (4,129 participants) were included. Massage, electroacupuncture, Baduanjin and acupuncture significantly reduced fasting plasma glucose (FPG) compared with lifestyle interventions. Massage (SUCRA: 99.3%) was the most effective in FPG reduction, followed by electroacupuncture, Baduanjin, acupuncture. All therapies significantly improved 2-hour postprandial plasma glucose (2hPG), among them, massage (SUCRA: 94.1%) was the best, followed by electroacupuncture, Taiji, acupuncture, Baduanjin and ACE. Acupuncture (SUCRA: 81.8%) was the only intervention that significantly reduced HbA1c. Furthermore, electroacupuncture, massage and acupuncture significantly reduced body mass index (BMI), with electroacupuncture (SUCRA: 98.8%) the best, followed by massage and acupuncture. Electroacupuncture and Baduanjin significantly reduced total cholesterol, with electroacupuncture (SUCRA: 79.7%) the best, Baduanjin the second. ACE and Baduanjin significantly reduced triglycerides, with ACE (SUCRA: 74.9%) the best, Baduanjin the second.

**Conclusion:**

NPTTCM may provide potential benefits for improving metabolic outcomes in prediabetes. Massage showed a favorable ranking for reducing FPG and 2hPG, while acupuncture showed a favorable ranking for HbA1c reduction. Electroacupuncture and ACE showed potential benefits for BMI and lipid management. However, given the limitations, these results should be interpreted with caution. Future head-to-head and rigorously designed studies are needed to provide further evidence.

**Systematic Review Registration:**

https://www.crd.york.ac.uk/prospero/display_record.php?RecordID=1129592, identifier CRD420251129592.

## 1 Introduction

Prediabetes is defined as a state of impaired glucose metabolism, including impaired fasting glucose (IFG) and impaired glucose tolerance (IGT) (1). Latest International Diabetes Federation statistics indicate that morbidity of IFG is 9.2% and IGT 12.0% (2), with an annual progression rate to diabetes of 5-10% and a lifetime progression risk as high as 74% (3). Furthermore, prediabetes is associated with a 13% increased risk of all-cause mortality and predisposes individuals to cardiovascular diseases, microvascular complications, chronic kidney disease, and various malignancies, including liver and colorectal cancers (4, 5). Notably, these complications may arise prior to the formal diagnosis of diabetes (6). Therefore, timely and effective interventions at this stage are crucial.

Lifestyle interventions (LI) represent primary interventions for prediabetes (7). However, their long-term adherence remains challenging for many patients. In a 21-year follow-up study, despite a significant reduction in diabetes incidence during the initial intervention period, the cumulative incidence of diabetes in the intervention group gradually converged with that of the control group over time (8). For those who fail LI or are at high risk, pharmacological treatments may be the choice. However, no drug has an indication for the treatment of prediabetes. In recent years, non-pharmacological therapies of traditional Chinese medicine (NPTTCM) have demonstrated significant potential in type 2 diabetes mellitus (T2DM) prevention, treatment, and complication therapies (9, 10). Several meta-analyses further indicated that interventions such as acupuncture-related therapies and traditional exercises could improve glycemic and lipid profiles in prediabetes and reduce the risk of disease progression (11–13). NPTTCM are characterized by their simplicity, practicality, and cost effectiveness, making them particularly suitable for primary care.

However, there is not any network meta-analysis (NMA) to systematically evaluate the efficacies of different NPTTCM in prediabetes. To bridge the gap, we designed this study to evaluate the effects of different NPTTCM on body mass index (BMI), glycemic and lipid control for prediabetes.

## 2 Methods

### 2.1 Study design

This work was conducted by Preferred Reporting Items for Systematic Reviews and Meta-Analyses for Network Meta-Analyses (14). The protocol was registered in the International Prospective Register of Systematic Reviews database (CRD420251129592).

### 2.2 Search strategy

A comprehensive search was conducted in PubMed, Embase, Web of Science, Cochrane Library, the Chinese National Knowledge Infrastructure, the Chinese Science and Technology Journals Database, the Wanfang Database and Sinomed. These databases were searched from their inception until July, 26, 2025. No language or time limitations were applied. The search comprised MeSH terms with all subheadings, along with relevant free-text terms related to “prediabetic state”, “acupuncture”, “acupoint catgut embedding”, “massage”, “Baduanjin”, “Taiji” and so on (**Supplementary Table 1**).

### 2.3 Inclusion criteria

Studies that met the following criteria were included. (1) Type of studies: Randomized controlled trials (RCTs) were included. (2) Type of participants: This study included research recruiting participants aged ≥18 years with prediabetes, without sex, or race restrictions. The diagnosis of prediabetes was based on the American Diabetes Association (ADA) criteria (15), World Health Organization (WHO) criteria (16) and the Chinese Diabetes Society (CDS) Guidelines for the Prevention and Treatment of T2DM (17). (3) Type of interventions: The treatment group was treated with NPTTCM. The control group was treated with LI, such as health education, routine care and lifestyle intervention. (4) Type of outcomes: Glycosylated hemoglobin (HbA1c), fasting plasma glucose (FPG), postprandial 2-hour postprandial plasma glucose (2hPG), total cholesterol (TC), triglycerides (TG), BMI. The intervention duration in studies incorporating HbA1c should exceed 8 weeks to meet the base time for HbA1c changes.

### 2.4 Exclusion criteria

Studies that met any of the following criteria were excluded. (1) Review articles, case reports, animal and cell experiments and all other non-RCTs. (2) Studies with incomplete general data and baseline indicators. Researchers didn’t get reply after contacting with the author. (3) Studies whose full text could not be obtained through public databases or other sources. (4) Interventions involving pharmacological treatments. The control group did not undergo any intervention. (5) Studies included patients diagnosed with diabetes, severe hypertension, severe hepatic or renal insufficiency, severe cardiovascular disease, other potentially life-threatening conditions, patients with a confirmed diagnosis of mental illness, and pregnant or breastfeeding women.

### 2.5 Study selection and data extraction

Using the reference manager Endnote X9, citations were imported, and duplicates were excluded. Two authors independently conducted title and abstract screening, and full-text screening to identify relevant and potentially relevant publications. The following information from the included studies was extracted by two authors: (1) study information including publication year, first author; (2) participants including sample size, age, sex, and diagnostic criteria; including the type of therapy and duration of treatment; (3) and outcome measures. If data were missing, we would email the corresponding author to obtain the relevant data. Any disagreements were resolved through discussion and consensus, with the third author reviewing and making the final decision.

### 2.6 Assessment of risk of bias

Two authors assessed the quality of the literature using the Risk of Bias 2.0 tool (18). Examined domains: (1) randomization process; (2) deviations from the intended interventions; (3) missing outcome data; (4) measurement of the outcome; (5) selection of the reported results.

### 2.7 Statistical analysis

Software RevMan 5.4.1 was used to directly compare the various treatments in outcomes. We used box plots to evaluate the transitivity assumption by comparing the distribution of potential effect modifiers (mean age, percentage of male, sample size, baseline severity, intervention duration and publication year) across the studies. The mean differences (MD) with 95% confidence intervals (CI) for continuous outcomes, heterogeneity quantified as high with I² values>50% and P<0.05. If substantial heterogeneity existed, a random-effects model was used to pool measures; otherwise, a fixed-effects model was used. The potential sources of heterogeneity were explored by meta-regression. The NMA was based on the frequency model and performed by using software STATA 15.0. The normal likelihood was employed to conduct all analyses of continuous outcomes. A network evidence diagram was generated to illustrate the comparisons among the interventions. The necessity for loop inconsistency detection was determined by the presence of closed loops between the interventions. The NMA was performed using forest plots and ranking tables. To rank the treatments for each outcome, we used the surface under the cumulative ranking curve (SUCRA) and the mean ranks. We used comparison-adjusted funnel plots to identify evidence for small sample effects.

## 3 Results

### 3.1 Study selection

**Figure 1:**
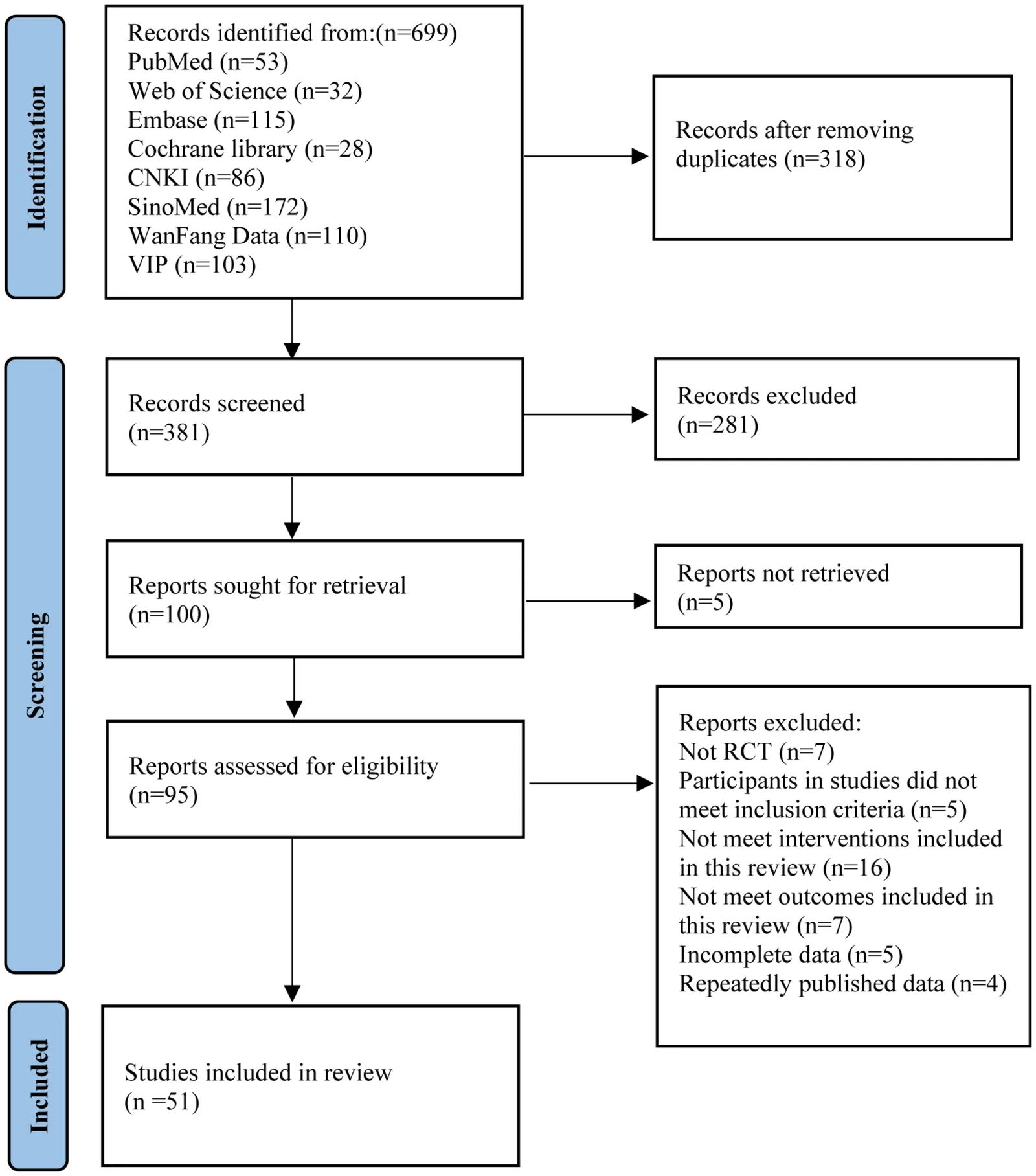
Study selection process.

The initial search yielded 699 records, of which 318 duplicate publications were eliminated. After screening the titles and abstracts, 281 articles were excluded. 95 studies were selected for full-text assessment during the title and abstract screening. Ultimately, 51 RCTs were deemed relevant (**Figure 1**).

### 3.2 Study characteristics

Among the 51 enrolled RCTs (19–69), 16 were on acupuncture, 9 on acupoint catgut embedding (ACE), 13 on Baduanjin, 4 on electroacupuncture, 4 on massage and 5 on Taiji. Totally 4129 participants were included, with 46.29% males, the average age were 50.35 ± 10.06 years (**Supplementary Table 2**).

### 3.3 Risk of bias assessment

Overall, 14 (27.5%) of 51 trials were rated as high risk of bias, 35 (68.6%) trials as moderate, and 2 (3.9%) as low (**Supplementary Table 3**). The main sources of potential bias were related to deviations from intended interventions and the randomization process. Specifically, 11 studies were evaluated as high risk of deviations from intended interventions. In addition, 25 studies were judged with “some concerns” for the unclear description of the generation of random sequences. No study was rated as high risk due to issues in the randomization process.

### 3.4 Evaluation of transitivity

The box plots showed that, despite some variability across treatment comparisons, the distributions of the assessed effect modifiers overlapped to some extent (**Supplementary Figures 1**–**8**). These findings suggested that no major imbalance in the measured effect modifiers was apparent. However, the possibility of residual imbalance due to unmeasured effect modifiers cannot be completely excluded.

### 3.5 Evaluation of heterogeneity

High and statistically significant overall heterogeneity was observed across all outcomes (P<0.001), with I^2^ values as follows: BMI (94.7%), FPG (92.6%), 2hPG (91.1%), HbA1c (91.1%), TG (89.6%), and TC (75.2%) (**Supplementary Tables 4**–**9**). To explore potential sources of heterogeneity, meta-regression was performed using intervention frequency, duration, sample size and mean age as covariates (**Supplementary Tables 10**–**15**). The results indicated that neither covariate significantly predicted the variation in the effect size.

### 3.6 Evidence network

**Figure 2:**
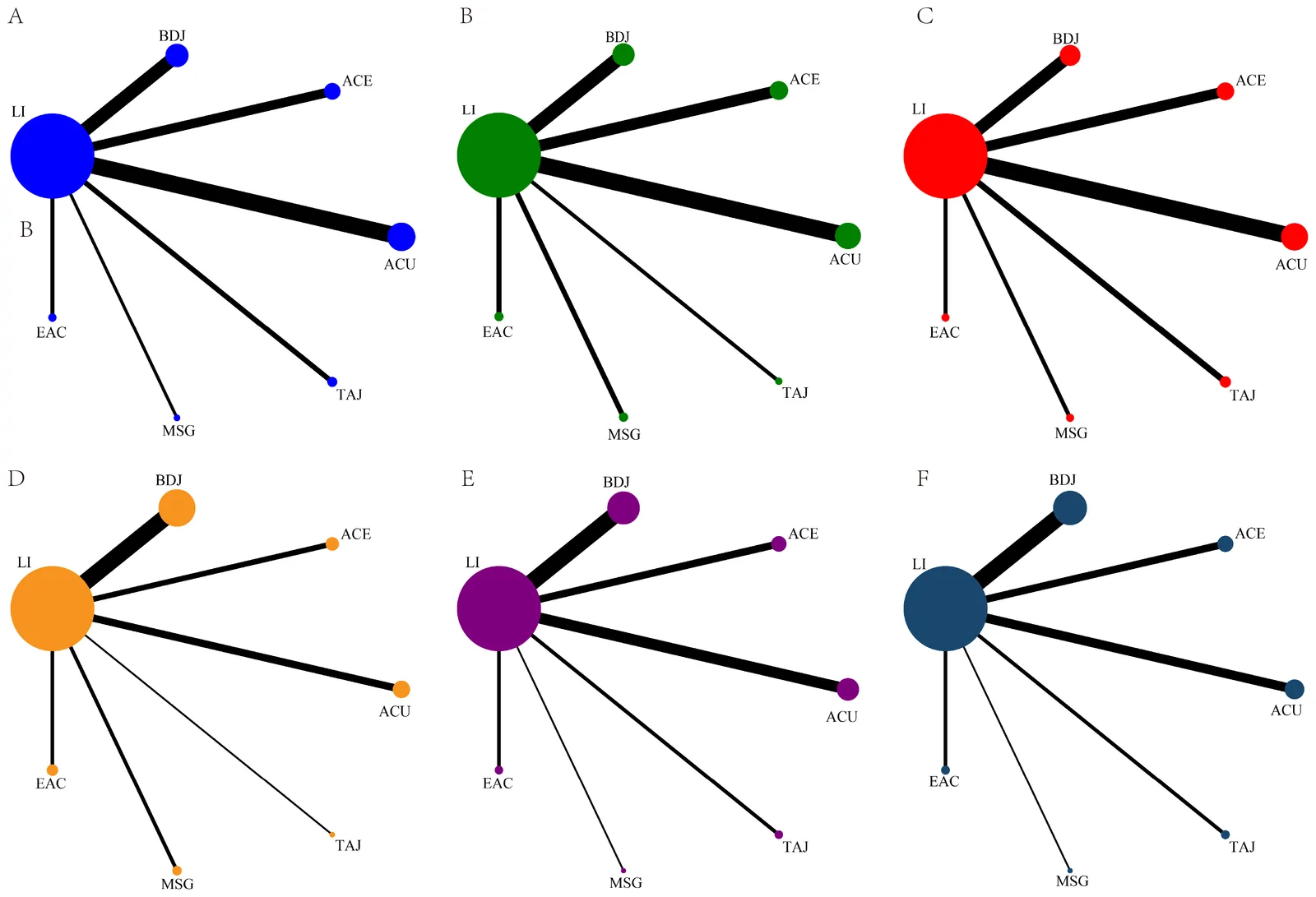
The network plots of treatment effects for all outcomes. **(A)** FPG, **(B)** 2hPG, **(C)** HbA1c, **(D)** BMI, **(E)** TC, **(F)** TG. ACU, acupuncture; ACE, acupoint catgut embedding; BDJ, Baduanjin; LI, lifestyle intervention; EAC, electroacupuncture; MSG, massage; TAJ, Taiji.

Each node represents a distinct intervention, and the size of both nodes and connecting lines in the graph is proportional to the number of studies. The network revealed no closed loops (**Figure 2**). For FPG, 50 studies (98.0%) were included, with 100 arms and 4,046 patients (2,027 in the experimental group and 2,019 in the control group). Acupuncture vs LI was the most frequently compared intervention (16 studies), followed by ACE vs LI (9 studies), Baduanjin vs LI (13 studies), electroacupuncture vs LI care (4 studies), massage vs LI (3 studies), and Taiji vs LI (5 studies). For 2hPG, 44 studies (86.3%) were included, with 88 arms and 3,704 patients (1,854 experimental, 1,850 control). The most common comparison was acupuncture vs LI (13 studies), followed by ACE (9 studies), Baduanjin (11 studies), electroacupuncture (4 studies), massage (4 studies), and Taiji (3 studies). For HbA1c, 26 studies (51.0%) were included, with 52 arms and 1,478 patients (745 experimental, 733 control). Acupuncture vs LI remained the most represented comparison (8 studies), followed by ACE (5 studies), Baduanjin (6 studies), electroacupuncture (2 studies), massage (2 studies), and Taiji (3 studies). For BMI, 21 studies (41.2%) were included, with 42 arms and 2,058 patients (1,029 experimental, 1,029 control). Baduanjin vs LI constituted the most frequent comparison (9 studies), followed by acupuncture (4 studies), ACE (3 studies), electroacupuncture (2 studies), massage (2 studies), and Taiji (1 study). For TC, 24 studies (47.1%) were included, with 48 arms and 1,962 patients (982 experimental, 980 control). Baduanjin vs LI was the most common comparison (9 studies), followed by acupuncture (6 studies), ACE (4 studies), electroacupuncture (2 studies), massage (1 study), and Taiji (2 studies). For TG, 23 studies (45.1%) were included, with 46 arms and 1,907 patients (955 experimental, 952 control). Baduanjin vs LI was the most frequent comparison (9 studies), followed by acupuncture (5 studies), ACE (4 studies), electroacupuncture (2 studies), massage (1 study), and Taiji (2 studies).

### 3.7 Adverse events

Of the 51 studies, 12 (23.5%) addressed safety outcomes (**Table 1**). Seven studies reported no adverse events (AEs) in both groups, while 5 studies reported a total of 174 AEs. ACE was associated with the highest number of reported AEs (171 cases), largely attributable to a study which recorded 168 cases of mild reactions, including gastrointestinal discomfort, emotional distress, and localized pain or swelling. The AEs of acupuncture included hematoma and subcutaneous congestion. Overall, AEs were transient and manageable, with no reports of serious AEs. However, due to the limited reporting of AEs in the included studies, the safety profile of the NPTTCM could not be fully established.

**Table 1:** Occurrence of adverse reactions.

Study ID	Therapy	Adverse events		Response
		Treatment group	Control group	
Li Y 2023 (54)	ACU VS LI	0 case	0 case	
Gong ZF 2024 (66)	ACU VS LI	0 case	0 case	
Zhang H 2022 (22)	ACU VS LI	Hematoma (2 cases)	0 case	The hematoma subsided after pressing treatment in time
Tan CJ 2019 (33)	ACU VS LI	0 case	0 case	
Hu ZB 2024 (63)	ACU VS LI	Subcutaneous congestion (1 case)	0 case	Subcutaneous congestion recovered completely in about a week
Qu FZ 2016 (41)	ACE VS LI	Low fever (2 cases)	0 case	Give physical cooling and drink plenty of warm boiled water to relieve after medical advice
Zhang LB 2010 (21)	ACE VS LI	0 case	0 case	
Wang Y 2021 (29)	ACE VS LI	Pain is intolerable (1case)	0 case	Withdrawal from the trial
Xue N 2017 (25)	ACE VS LI	Loss of appetite (42 cases), postprandial epigastric pain (28 cases), noisy gastral cavity (5 cases), emotional distress (32 cases), insomnia (6 cases), mental hyperfunction (3 cases), local pain and swelling (14 cases), local swelling and fever (7 cases), local skin itching (2 cases), whole body muscle soreness (10 cases), low fever (8 cases), hypodynamia (3 cases), constipation (2 cases), chest distress (2 cases), back stiffness and pain (1 case), palpitation (1 case), menostaxis (1 case), tissue reactions to sutures (1 case)	0 case	
Li YM 2013 (52)	ACE VS LI	0 case	0 case	
Ma XJ 2022 (43)	BDJ VS LI	0 case	0 case	
Ren QJ 2022 (39)	BDJ VS LI	0 case	0 case	

ACU, acupuncture; ACE, acupoint catgut embedding; BDJ, Baduanjin; LI, lifestyle intervention; EAC, electroacupuncture; MSG, massage, TAJ, Taiji.

### 3.8 NMA efficacy outcomes

For FPG, the forest plot showed that massage (MD: –1.49; 95%CI: [–2.14, –0.84]), electroacupuncture (MD: –0.72; 95%CI: [–1.20, –0.24]), Baduanjin (MD: –0.50; 95%CI: [–0.78, –0.22]) and acupuncture (MD: –0.45; 95%CI: [–0.69, –0.20]) significantly reduced FPG compared with LI. Massage was significantly superior to acupuncture, ACE, Baduanjin, and Taiji in improving FPG, no other significant differences were observed between other pairs of therapies. Probability ranking results were as follows: massage (SUCRA: 99.3%) > electroacupuncture (SUCRA: 73.7%) > Baduanjin (SUCRA: 55.8%) > acupuncture (SUCRA: 49.3%) > Taiji (SUCRA: 43.1%) > ACE (SUCRA: 26.9%) > LI (SUCRA: 2.0%).

For 2hPG, compared to LI, massage (MD: –1.80; 95%CI: [–2.63, –0.96]), electroacupuncture (MD: –1.37; 95%CI: [–2.02, –0.72]), Taiji (MD: –0.95; 95%CI: [–1.87, –0.04]), acupuncture (MD: –0.87; 95%CI: [–1.23, –0.51]), Baduanjin (MD: –0.86; 95%CI: [–1.29, –0.43]) and ACE (MD: –0.53; 95%CI: [–0.95, –0.11]) showed a significant reduction in 2hPG. Massage was significantly superior to acupuncture and ACE, while electroacupuncture was superior to ACE. No other pairwise comparisons reached statistical significance. Probability ranking results were as follows: massage (SUCRA:94.1%) > electroacupuncture (SUCRA:79.5%) > Taiji (SUCRA:54.1%) > acupuncture (SUCRA:49.3%) > Baduanjin (SUCRA:48.2%) > ACE (SUCRA:24.2%) > LI (SUCRA:0.4%).

For HbA1c, among all therapies, only acupuncture (MD: –0.80; 95%CI: [–1.27, –0.32]) significantly reduced HbA1c compared with LI. No statistically significant differences were found in any pairwise comparisons between therapies. Probability ranking results were as follows: acupuncture (SUCRA:81.8%) > ACE (SUCRA:66.4%) > Taiji (SUCRA:54.2%) > massage (SUCRA:51.8%) > electroacupuncture (SUCRA:47.3%) > Baduanjin (SUCRA:34.0%) > LI (SUCRA:14.4%).

For BMI, acupuncture (MD: –2.44; 95%CI: [–3.92, –0.96]), electroacupuncture (MD: –5.82; 95%CI: [–8.00, –3.63]), and massage (MD: –3.31; 95%CI: [–5.68, –0.95]) significantly reduced BMI compared with LI, while ACE, Baduanjin, and Taiji showed no significant effects. Electroacupuncture was superior to acupuncture, ACE, Baduanjin, and Taiji. Probability ranking results were as follows: electroacupuncture (SUCRA:98.8%) > massage (SUCRA:78.4%) > acupuncture (SUCRA:68.7%) > Baduanjin (SUCRA:38.6%) > ACE (SUCRA:27.7%) > Taiji (SUCRA:23.3%) > LI (SUCRA:14.6%).

For TC, Baduanjin (MD: –0.30; 95%CI: [–0.57, –0.04]) and electroacupuncture (MD: –0.54; 95%CI: [–1.07, –0.01]) significantly reduced TC compared with LI. No significant differences were observed in pairwise comparisons between therapies. Probability ranking results were as follows: electroacupuncture (SUCRA:79.7%) > ACE (SUCRA:65.1%) > Baduanjin (SUCRA:56.8%) > Taiji (SUCRA:53.4%) > massage (SUCRA:41.4%) = acupuncture (SUCRA:41.4%) > LI (SUCRA:12.2%).

For TG, ACE (MD: –0.41; 95%CI: [–0.80, –0.03]) and Baduanjin (MD: –0.31; 95%CI: [–0.57, –0.05]) significantly reduced TG compared with LI, while no significant differences were found between therapy pairs. Probability ranking results were as follows: ACE (SUCRA:74.9%) > Baduanjin (SUCRA:64.9%) > massage (SUCRA:59.8%) > Taiji (SUCRA:55.6%) > electroacupuncture (SUCRA:43.3%) > acupuncture (SUCRA:34.6%) > LI (SUCRA:17.0%).

**Figure 3:**
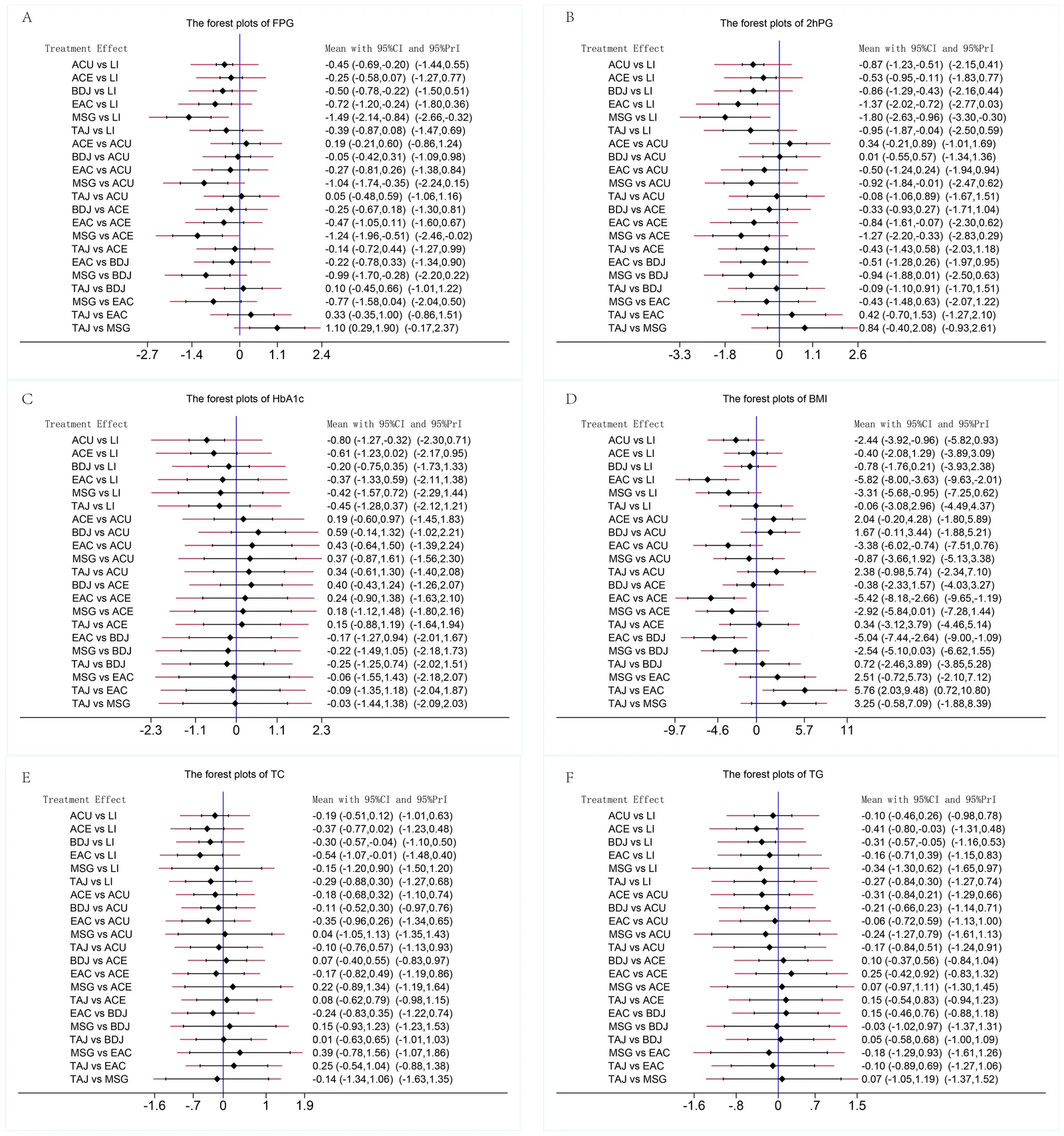
The forest plots of network meta-analysis for all outcomes. **(A)** FPG, **(B)** 2hPG, **(C)** HbA1c, **(D)** BMI, **(E)** TC, **(F)** TG. ACU, acupuncture; ACE, acupoint catgut embedding; BDJ, Baduanjin; LI, lifestyle intervention; EAC, electroacupuncture; MSG, massage; TAJ, Taiji.

The detailed outcomes of comparison among 6 NPTTCM were shown in **Figure 3** and **Supplementary Tables 16**–**21**. The ranking of treatments based on cumulative probability plots and SUCRAs of NPTTCM are shown in **Supplementary Tables 22**–**27** and **Supplementary Figures 9**–**14**.

### 3.9 Publication bias

The comparison-adjusted funnel plot was drawn for each outcome included in the study (**Supplementary Figure 15**). The funnel plots for 2hPG, HbA1c, BMI, and TC each show several points located on both sides and at the bottom. The resulting asymmetric distribution of points indicates potential publication bias or small sample effects. The funnel plots for FPG and TG showed symmetrical distributions of studies around the midline, suggesting a low probability of publication bias or small-sample effects.

## 4 Discussion

This NMA included 51 RCTs involving 4,129 patients randomly assigned to 6 NPTTCM or LI. Our NMA suggested that massage was most likely to rank first for reducing FPG and 2hPG, while acupuncture was most likely to perform best at reducing HbA1c. For BMI and TC, electroacupuncture showed the highest ranking probability for improvement, whereas ACE was superior for TG reduction. However, these comparative rankings should be interpreted cautiously considering the limitations of the available evidence.

Acupuncture demonstrated significant improvements in reducing FPG, 2hPG, HbA1c and BMI compared with LI, but no significant effects were observed on TC or TG. Electroacupuncture effectively reduced FPG, 2hPG, BMI, and TC, and was significantly superior to acupuncture in improving BMI. The differences between electroacupuncture and manual acupuncture may partly reflect variations in stimulation characteristics and their downstream biological responses. Previous experimental studies have suggested that acupuncture may improve glucose metabolism primarily through enhancing insulin sensitivity and regulating insulin signaling pathways. In rat model of insulin resistance (IR), acupuncture has been reported to restore the expression of insulin signaling-related molecules, including insulin receptor substrate (IRS)-1, IRS-2, protein kinase B (Akt2), and glucose transporter type (GLUT) 4 to normal, activating the signal transduction pathway of the phosphatidylinositol 3-kinase (PI3K)/Akt (70). Compared with manual acupuncture, electroacupuncture provides additional electrical stimulation, which may result in different physiological responses depending on stimulation parameters. Electroacupuncture has been suggested to exert additional effects on lipid metabolism through distinct regulatory pathways. Electroacupuncture might effectively promote white adipose tissue browning through activation of glucagon-like peptide (GLP)-1 neurons in the solitary nucleus, the promotion of adenosine 5’-monophosphate-activated protein kinase (AMPK) phosphorylation in the hypothalamic arcuate nucleus, the upregulation of uncoupling protein 1 (71), possibly through activation of the AMPK/sirtuin (SIRT)1 signaling pathway and increased neuregulin 4 levels (72).

ACE demonstrated potential benefits in reducing 2hPG and TG, but no significant effect was found on FPG, HbA1c, BMI and TC. Unlike a previous meta-analysis in this field (73), our analysis did not find a significant effect of ACE on FPG and HbA1c, but we included a greater number of studies and participants. This discrepancy may be related to differences in study inclusion criteria, intervention characteristics, and the number of included studies. In particular, we excluded studies involving combined drug therapy to independently evaluate the efficacy of ACE. Mechanistically, ACE may regulate the peroxisome proliferator-activated receptors (PPAR) signaling pathway by upregulating lipoprotein lipase expression and downregulating solute carrier family 27 member 2, fatty acid binding protein 1, and apolipoprotein c3 expression, thereby further improving body fat metabolism (74). In addition, ACE may activate the PI3K/Akt signaling pathway, which could contribute to improved glucose and lipid metabolic homeostasis (75).

Massage may offer advantages in improving FPG and 2hPG, a finding consistent with previous guidelines (76). However, limited by the number and quality of RCTs, there is insufficient evidence to recommend this regimen as the optimal treatment option. Moreover, our analysis suggested a potential benefit of massage in reducing BMI. Massage has been reported to reduce food intake and body weight in obese rats through modulation of the G protein-coupled receptor (GPR)41/GPR43-peptide YY/GLP-1 signaling axis (77). A study shows that massage could improve IR by regulating the secretion of adipocytokines, pro-inflammatory cytokines as well as AMPK/SIRT1/peroxisome proliferator-activated receptor gamma coactivator-1α signaling pathways in the skeletal muscle of rat (78). Another study has indicated that massage may enhance fatty acid oxidation, lipid metabolism, and glucose homeostasis through activation of the PPARγ signaling pathway in white adipose tissue (79). However, we did not observe significant efficacy of massage in improving HbA1c, which may be attributable to the short intervention duration (58). Furthermore, previously published meta-analyses have not addressed the impact of massage on HbA1c in patients with diabetes (80).

Our analysis found that Baduanjin significantly improved FPG, 2hPG, TC, and TG, while Taiji demonstrated a beneficial effect on 2hPG. A meta-analysis encompassing 5 traditional Chinese exercises indicated that Baduanjin possesses the strongest capability for glycemic regulation (81). However, a more recent meta-analysis presented a different conclusion, reporting that Taiji demonstrated the greatest improvements in glycemic outcomes, while Baduanjin exhibited the most significant effects on lipid parameters (11). Notably, these studies show intervention duration, treatment course, and supervised status as key determinants of efficacy, with significant lipid improvements often requiring longer interventions, which may explain the divergent findings across trials. Baduanjin and Taiji are characterized by coordinated low-to-moderate intensity movements combined with breath regulation, relaxation, and meditative attention. These exercises may reduce cortisol secretion and modulate sympathetic nervous system activity, thereby potentially improving insulin sensitivity (82, 83). Taiji may further influence metabolic regulation through multiple potential mechanisms, including enhanced insulin sensitivity (via PI3K/Akt pathway and GLUT4 translocation), attenuation of oxidative stress and β-cell dysfunction, and improvement of endothelial function through modulation of inflammatory and vascular responses (84).

In terms of safety, although only 12 (23.5%) studies explicitly reported safety outcomes, most documented AEs were mild, transient, and manageable. Notably, the safety of ACE warrants closer attention, as it accounted for most reported incidents. This higher frequency of AEs is likely attributable to the minimally invasive nature of ACE and potential tissue responses to the suture material (85). Acupuncture and Baduanjin exhibited a low incidence of AEs, while the safety outcomes for other interventions were not systematically document. Although our findings support the reasonable safety of NPTTCM, the paucity of standardized safety reporting across trials remains a limitation. Therefore, future large-scale studies should prioritize rigorous, standardized monitoring of AEs to provide a more definitive safety assessment for these therapies.

Overall, the metabolic effects of NPTTCM may involve multiple pathways related to insulin sensitivity, lipid metabolism and neuroendocrine regulation. However, these mechanisms have not yet been sufficiently validated in large-scale clinical studies and should be regarded as biologically plausible rather than definitive explanations for the observed effects.

## 5 Limitations and prospect

Several limitations of this study should be acknowledged. First, most studies (68.6%) exhibited a moderate or somewhat uncertain risk of bias. Due to the nature of NPTTCM, blinding of participants was often challenging. Few trials attempted to blind outcome assessors to minimize potential methodological bias. Consequently, most included RCTs are susceptible to performance and detection bias. Although lack of allocation concealment and blinding may be associated with exaggerated effect estimates for subjective outcomes, the degree of bias seems to be rather limited (86).

Second, high heterogeneity was observed across all outcomes, which remained largely unexplained by our meta-regression on intervention frequency, duration sample size and age. This variability may stem from unmeasured factors such as practitioner expertise, specific intervention nuances, concomitant lifestyle advice, and so on.

Third, the reliability of the findings may have been impacted by the absence of direct comparisons between different NPTTCM and the fact that the evidence network did not form closed loops. Additionally, some studies had small sample sizes. This renders the mean values and MD susceptible to the influence of individual outliers and more likely to reflect spuriously high or low effects due to chance.

Fourth, although NPTTCM appeared to be provisionally safe based on the available reports, the limited reporting of AEs in the included studies precludes a reliable assessment of their safety profile. Further confirmation through more rigorously designed trials with standardized safety monitoring is warranted.

Finally, we excluded studies involving participants receiving concomitant pharmacological therapies. Although this criterion may strengthen the plausibility of the transitivity assumption by reducing heterogeneity, it may also limit the applicability of our findings to real-world clinical settings where NPTTCM are frequently used as adjunctive therapies alongside conventional medical management. Therefore, the additional benefits of NPTTCM when combined with modern therapies in real-world clinical settings remain uncertain. Future well-designed studies are warranted to investigate the effectiveness and safety of NPTTCM combined with modern medical treatments.

In summary, the clinical applicability of these results is currently tempered by several limitations inherent in the evidence base. Consequently, the estimated effect sizes should be interpreted with caution. Future research should prioritize rigorously designed, large-scale RCTs from diverse cultural backgrounds conducting direct comparisons between these therapies. In addition, future studies should focus on long-term clinical outcomes and AEs associated with NPTTCM in individuals with prediabetes. Real-world studies are also warranted to identify patient characteristics associated with better treatment responses and to explore optimal management strategies combining NPTTCM with modern medical approaches, thereby providing evidence for precision-based clinical practice. Furthermore, clinical trial protocols should be registered prospectively according to the internationally recognized SPIRIT statement, and study results should be reported in accordance with the CONSORT guidelines.

## 6 Conclusions

In conclusion, NPTTCM may provide potential benefits for improving metabolic outcomes in individuals with prediabetes. Different NPTTCM showed potentially favorable effects on different outcomes. Massage showed a preferable improvement in FPG and 2hPG. Acupuncture showed a relatively favorable ranking for HbA1c reduction, while electroacupuncture and ACE demonstrated potentially beneficial effects on BMI, TC, and TG. However, given the limitations of the current evidence base, including potential risk of bias, heterogeneity, and limited direct comparisons among interventions, these results should be interpreted with caution, and no specific NPTTCM can currently be considered superior. Future high-quality, large-scale, head-to-head trials are warranted to further validate the comparative safety and efficacy of these NPTTCM.

## Data Availability

The original contributions presented in the study are included in the article/**Supplementary Material**. Further inquiries can be directed to the corresponding author.
