# Supplementary material for: Non-pharmacological therapies of traditional Chinese medicine in prediabetes: a systematic review and network meta-analysis of randomized controlled trials
Source: Front Endocrinol (Lausanne). 2026 Aug 27;17:1907358. doi: 10.3389/fendo.2026.1907358 (PMC13558012; doi:10.3389/fendo.2026.1907358)
Supplement: Supplementary file 1 [file SupplementaryFile1.docx]

***Supplementary Material***

**Appendix 1. Retrieval strategies**

| **Supplementary Table 1.** **Retrieval strategies** | | |
| --- | --- | --- |
| **Database** | **Number** | **Search terms** |
| **PubMed** | #1 | (Prediabetic State[MeSH Terms]) OR (Prediabetic States[Title/Abstract]) OR (State, Prediabetic[Title/Abstract]) OR (States, Prediabetic[Title/Abstract]) OR (Prediabetes[Title/Abstract]) OR (Glucose Intolerance[MeSH Terms]) OR (Glucose Intolerances[Title/Abstract]) OR (Intolerance, Glucose[Title/Abstract]) OR (Intolerances, Glucose[Title/Abstract]) OR (Impaired Glucose Tolerance[Title/Abstract]) OR (Glucose Tolerance, Impaired[Title/Abstract]) OR (Glucose Tolerances, Impaired[Title/Abstract]) OR (Impaired Glucose Tolerances[Title/Abstract]) OR (Tolerance, Impaired Glucose[Title/Abstract]) OR (Tolerances, Impaired Glucose[Title/Abstract]) OR (impaired fasting glucose[Title/Abstract]) |
|  | #2 | (Acupuncture[MeSH Terms]) OR (Pharmacopuncture[Title/Abstract]) OR (Moxibustion[MeSH Terms]) OR (Moxabustion[Title/Abstract]) OR (Acupoint catgut embedding[Title/Abstract]) OR (Massage[MeSH Terms]) OR (Zone Therapy[Title/Abstract]) OR (Therapies, Zone[Title/Abstract]) OR (Zone Therapies[Title/Abstract]) OR (Therapy, Zone[Title/Abstract]) OR (Massage Therapy[Title/Abstract]) OR (Massage Therapies[Title/Abstract]) OR (Therapies, Massage[Title/Abstract]) OR (Therapy, Massage[Title/Abstract]) OR (Tuina[Title/Abstract]) OR (Tai Ji[MeSH Terms]) OR (Tai-ji[Title/Abstract]) OR (Tai Chi[Title/Abstract]) OR (Chi, Tai[Title/Abstract]) OR (Tai Chi Chuan[Title/Abstract]) OR (Taiji[Title/Abstract]) OR (Taijiquan[Title/Abstract]) OR (T'ai Chi[Title/Abstract]) OR (Tai Ji Quan[Title/Abstract]) OR (Ji Quan, Tai[Title/Abstract]) OR (Quan, Tai Ji[Title/Abstract]) OR (Baduanjin[Title/Abstract]) OR (Eight Brocade[Title/Abstract]) OR (eight brocades[Title/Abstract]) OR (Wuqinxi[Title/Abstract]) OR (Yijinjing[Title/Abstract]) OR (Shaolin Neigong[Title/Abstract]) |
|  | #3 | #1 AND #2 |
| **EMBASE** | #1 | 'impaired glucose tolerance'/exp OR 'glucose intolerance'/exp |
|  | #2 | 'chemical diabetes':ti,kw,ab OR 'chemical diabetes mellitus':ti,kw,ab OR 'diabetes mellitus, potential':ti,kw,ab OR 'diabetes, chemical':ti,kw,ab OR 'diabetes, latent':ti,kw,ab OR 'genetic prediabetes':ti,kw,ab OR 'glucose tolerance impairment':ti,kw,ab OR 'glucose tolerance, potentially impaired':ti,kw,ab OR 'impaired glucose tolerance, potential':ti,kw,ab OR 'latent diabetes':ti,kw,ab OR 'latent diabetes mellitus':ti,kw,ab OR 'potential diabetes':ti,kw,ab OR 'potential diabetes mellitus':ti,kw,ab OR 'potential glucose tolerance impairment':ti,kw,ab OR 'pre diabetes mellitus':ti,kw,ab OR 'pre-clinical diabetes':ti,kw,ab OR 'pre-diabetes':ti,kw,ab OR 'preclinical diabetes':ti,kw,ab OR 'prediabetes':ti,kw,ab OR 'prediabetes mellitus':ti,kw,ab OR 'prediabetic stage':ti,kw,ab OR 'prediabetic state':ti,kw,ab OR 'subclinical diabetes':ti,kw,ab OR 'impaired glucose tolerance':ti,kw,ab |
|  | #3 | 'acupuncture'/exp OR 'acupuncture' OR 'catgut embedding'/exp OR 'catgut embedding' OR 'moxibustion'/exp OR 'moxibustion' OR 'massage'/exp OR 'massage' OR 'tai chi'/exp OR 'tai chi' |
|  | #4 | 'acupuncture therapy':ti,kw,ab OR 'shonishin':ti,kw,ab OR 'acupuncture':ti,kw,ab OR 'acet (acupoint catgut embedding therapy)':ti,kw,ab OR 'acupoint catgut embedding therapy':ti,kw,ab OR 'catgut implantation':ti,kw,ab OR 'catgut embedding':ti,kw,ab OR 'massage therapy':ti,kw,ab OR 'masso-therapy':ti,kw,ab OR 'massotherapy':ti,kw,ab OR 'sports massage':ti,kw,ab OR 'massage':ti,kw,ab OR 'tuina':ti,kw,ab OR 'tai chi chuan':ti,kw,ab OR 'tai ji':ti,kw,ab OR 'taiji quan':ti,kw,ab OR 'taijiquan':ti,kw,ab OR 'tai chi':ti,kw,ab OR 'baduanjin':ti,kw,ab OR 'eight brocade':ti,kw,ab OR 'eight brocades':ti,kw,ab OR 'wuqinxi':ti,kw,ab OR 'yijinjing':ti,kw,ab OR 'shaolin neigong':ti,kw,ab |
|  | #5 | #1 OR #2 |
|  | #6 | #3 OR #4 |
|  | #7 | #5 AND #6 |
| **Web of Science** | #1 | TS=("Prediabetic State*" OR "impaired fasting glucose" OR "State*, Prediabetic" OR "Prediabete*" OR "Glucose Intolerance*" OR "Intolerance*, Glucose" OR "Impaired Glucose Tolerance*" OR "Glucose Tolerance*, Impaired" OR "Tolerance*, Impaired Glucose") |
|  | #2 | TS=(“Acupuncture” OR "Pharmacopuncture" OR "Moxibustion" OR "Moxabustion" OR "Acupoint catgut embedding" OR "Massage" OR "Zone Therapy" OR "Therapies, Zone" OR "Zone Therapies" OR "Therapy, Zone" OR "Massage Therapy" OR "Massage Therapies" OR "Therapies, Massage" OR "Therapy, Massage" OR "Tuina" OR "Tai Ji" OR "Tai-ji" OR "Tai Chi" OR "Chi, Tai" OR "Tai Chi Chuan" OR "Taiji" OR "Taijiquan" OR "T'ai Chi" OR "Tai Ji Quan" OR "Ji Quan, Tai" OR "Quan, Tai Ji" OR "Baduanjin" OR " eight brocade*" OR "Wuqinxi" OR " Yijinjing" OR "Shaolin Neigong") |
|  | #3 | #1 AND #2 |
| **Cochrane Library** | #1 | MeSH descriptor: [Prediabetic State] explode all trees |
|  | #2 | MeSH descriptor: [Glucose Intolerance] explode all trees |
|  | #3 | (‘impaired fasting glucose’ or ‘Prediabetic States’ or ‘State, Prediabetic’ or ‘States, Prediabetic’ or ‘Prediabetes’ or ‘Glucose Intolerances’ or ‘Intolerance, Glucose’ or ‘Intolerances, Glucose’ or ‘Impaired Glucose Tolerance’ or ‘Glucose Tolerance, Impaired’ or ‘Glucose Tolerances, Impaired’ or ‘Impaired Glucose Tolerances’ or ‘Tolerance, Impaired Glucose’ or ‘Tolerances, Impaired Glucose’):ti,kw,ab |
|  | #4 | MeSH descriptor: [Acupuncture] explode all trees |
|  | #5 | MeSH descriptor: [Moxibustion] explode all trees |
|  | #6 | MeSH descriptor: [Massage] explode all trees |
|  | #7 | MeSH descriptor: [Tai Ji] explode all trees |
|  | #8 | ('Pharmacopuncture' or 'Moxabustion' or 'Acupoint catgut embedding' or ‘Zone Therapy’ or ‘Therapies, Zone’ or ‘Zone Therapies’ or ‘Therapy, Zone’ or ‘Massage Therapy’ or ‘Massage Therapies’ or ‘Therapies, Massage’ or ‘Therapy, Massage’ or ‘Tuina’ or ‘Tai-ji’ or ‘Tai Chi’ or ‘Chi, Tai’ or ‘Tai Chi Chuan’ or ‘Taiji’ or ‘Taijiquan’ or ‘T'ai Chi’ or ‘Tai Ji Quan’ or ‘Ji Quan, Tai’ or ‘Quan, Tai Ji’ or ‘Baduanjin’ or ‘Eight Brocade’ or ‘eight brocades’ or ‘Wuqinxi’ or ‘Yijinjing’ or ‘Shaolin Neigong’):ti,kw,ab |
|  | #9 | #1 OR #2 OR #3 |
|  | #10 | #4 OR #5 OR #6 OR #7 OR #8 |
|  | #11 | #9 AND #10 |
| **China National Knowledge Infrastructure** | #1 | （关键词：(糖尿病前期 + 空腹血糖受损 + 糖耐量受损)(精确)）AND（关键词：(针灸 + 针刺 + 艾灸 + 推拿 + 穴位埋线 + 太极拳 + 八段锦 + 易筋经 + 五禽戏 + 少林内功)(精确)） |
| **Wanfang Data** | #1 | 题名或关键词:((糖尿病前期 OR 空腹血糖受损 OR 糖耐量受损)) and 题名或关键词:((针灸 OR 针刺 OR 艾灸 OR 推拿 OR 穴位埋线 OR 太极拳 OR 八段锦 OR 易筋经 OR 五禽戏 OR 少林内功)) |
| **China Science and Technology Journal Database** | #1 | (((((((((((((((题名或关键词=糖尿病前期 OR 题名或关键词=prediabetes) OR 题名或关键词=prediabetic state) OR 题名或关键词=耐量异常) OR 题名或关键词=糖耐量低减) OR 题名或关键词=糖耐量减低) OR 题名或关键词=糖耐量降低) OR 题名或关键词=葡萄糖耐量受损) OR 题名或关键词=糖调节异常) OR 题名或关键词=糖耐量受损) OR 题名或关键词=糖调节受损) OR 题名或关键词=糖耐量减退) OR 题名或关键词=葡萄糖耐量减低) OR 题名或关键词=糖耐量异常) OR 题名或关键词=葡萄糖耐量异常) AND (((((((((题名或关键词=针灸 OR 题名或关键词=针刺) OR 题名或关键词=艾灸) OR 题名或关键词=推拿) OR 题名或关键词=穴位埋线) OR 题名或关键词=太极拳) OR 题名或关键词=八段锦) OR 题名或关键词=易筋经) OR 题名或关键词=五禽戏) OR 题名或关键词=少林内功)) |
| **SinoMed** | #1 | ( "糖尿病前期"[常用字段:智能] OR "空腹血糖受损"[常用字段:智能] OR "糖耐量受损"[常用字段:智能]) AND( "针灸"[常用字段:智能] OR "针刺"[常用字段:智能] OR "艾灸"[常用字段:智能] OR "推拿"[常用字段:智能] OR "穴位埋线"[常用字段:智能] OR "太极拳"[常用字段:智能] OR "八段锦"[常用字段:智能] OR "易筋经"[常用字段:智能] OR "五禽戏"[常用字段:智能] OR "少林内功"[常用字段:智能]) |

**Appendix 2. Assessment of the transitivity**

**Supplementary Figure 1.** Mean age distribution of the included study participants. Note: ACU: acupuncture, ACE: acupoint catgut embedding, BDJ: Baduanjin, LI: lifestyle intervention, EAC: electroacupuncture, MSG: massage, TAJ: Taiji.

**Supplementary Figure 2.** Percentage male distribution of the included study participants. Note: ACU: acupuncture, ACE: acupoint catgut embedding, BDJ: Baduanjin, LI: lifestyle intervention, EAC: electroacupuncture, MSG: massage, TAJ: Taiji.

**Supplementary Figure 3.** Sample size distribution of the included study participants. Note: ACU: acupuncture, ACE: acupoint catgut embedding, BDJ: Baduanjin, LI: lifestyle intervention, EAC: electroacupuncture, MSG: massage, TAJ: Taiji.

**Supplementary Figure 4.** Mean baseline FPG distribution of the included study participants. Note: ACU: acupuncture, ACE: acupoint catgut embedding, BDJ: Baduanjin, LI: lifestyle intervention, EAC: electroacupuncture, MSG: massage, TAJ: Taiji.

**Supplementary Figure 5.** Mean baseline 2hPG distribution of the included study participants. Note: ACU: acupuncture, ACE: acupoint catgut embedding, BDJ: Baduanjin, LI: lifestyle intervention, EAC: electroacupuncture, MSG: massage, TAJ: Taiji.

**Supplementary Figure 6.** Mean baseline HbA1c distribution of the included study participants. Note: ACU: acupuncture, ACE: acupoint catgut embedding, BDJ: Baduanjin, LI: lifestyle intervention, EAC: electroacupuncture, MSG: massage, TAJ: Taiji.

**Supplementary Figure 7.** Treatment duration distribution of the included study participants. Note: ACU: acupuncture, ACE: acupoint catgut embedding, BDJ: Baduanjin, LI: lifestyle intervention, EAC: electroacupuncture, MSG: massage, TAJ: Taiji.

**Supplementary Figure 8.** Publication year distribution of the included study participants. Note: ACU: acupuncture, ACE: acupoint catgut embedding, BDJ: Baduanjin, LI: lifestyle intervention, EAC: electroacupuncture, MSG: massage, TAJ: Taiji.

| **Appendix 3. Characteristics of trials**  **Supplementary** **Table 2.** Characteristics of trials included in this review ($\bar{\boldsymbol{x}}$±s) | | | | | | | | | | |
| --- | --- | --- | --- | --- | --- | --- | --- | --- | --- | --- |
| Study ID | Therapy  (T VS C) | Frequency/dosage | Diagnostic criteria | Sample size (T/C) | Male/Female (T) | Male/Female (C) | Age(T) | Age(C) | Outcomes | Treatment duration (month) |
| Li Y 2023(1) | ACU VS LI | 50 min, biw | WHO 1999 | 27/28 | 10/17 | 9/19 | 53.33 ± 6.02 | 54.50 ± 6.69 | ①④ | 3 |
| Gong ZF 2024(2) | ACU VS LI | 30 min, tiw | NM | 53/53 | 35/18 | 33/20 | 41.64±8.45 | 39.12±9.26 | ①②④⑤⑥ | 1 |
| Li Y 2020(3) | ACU VS LI | 45 min, tiw | CDS 2017 | 30/30 | 16/14 | 14/16 | 38.22±7.93 | 35.56±7.63 | ①② | 1 |
| Wang H 2013(4) | ACU VS LI | NM | WHO 1999 | 105/107 | 51/69 | 49/71 | 52.76±9.81 | 54.68±9.75 | ①②④⑤ | 1.5 |
| Zhu YP 2016(5) | ACU VS LI | qd | WHO 1999 | 160/160 | 56/104 | 61/99 | 52.87±9.92 | 54.79±9.86 | ①②④⑤⑥ | 6 |
| Luo GG 2019(6) | ACU VS LI | NM | CDS 2007 | 230/230 | 125/105 | 129/101 | 50.6±4.9 | 48.2±5.9 | ①②⑥ | 12 |
| Zhang H 2022(7) | ACU VS LI | 20 min, tiw | WHO 1999 | 30/30 | 12/18 | 15/15 | 61.37±9.97 | 58.57±9.92 | ①② | 3 |
| Tan CJ 2019(8) | ACU VS LI | 30 min, qod | CDS 2017 | 31/30 | 12/19 | 13/17 | 42.26± 9.91 | 42.33±8.49 | ①②③ | 3 |
| Ma SQ 2020(9) | ACU VS LI | qod | NM | 44/44 | 22/22 | 23/21 | 46.25±10.32 | 45.83±10.34 | ①② | 1 |
| Hu ZB 2024(10) | ACU VS LI | tiw | CDS 2017 | 29/26 | 6/23 | 7/19 | 49.62±11.06 | 48.42±12.79 | ①②③④⑤⑥ | 3 |
| Sun XJ 2018(11) | ACU VS LI | 30 min, qod | NM | 43/43 | 19/24 | 21/22 | 52±12 | 53±13 | ①②③ | 3 |
| Liu H 2020(12) | ACU VS LI | 30 min, tiw | NM | 41/41 | 28/13 | 29/12 | 55.7±6.2 | 55.2±6.4 | ①③ | 3 |
| Liu Y 2017(13) | ACU VS LI | 30 min, tiw | WHO 1999 | 32/28 | 15/17 | 14/14 | 55.4±6.7 | 52.7±5.8 | ①③④⑤ | 3 |
| Fan DS 2014(14) | ACU VS LI | 30 min | CDS 2004 | 32/30 | 22/10 | 21/9 | 43.16±1.03 | 43.07±1.08 | ①②③ | 3 |
| Lan W 2019(15) | ACU VS LI | 30 min, qod | CDS 2017 | 31/30 | 12/19 | 13/17 | 42.26±9.91 | 42.33±8.49 | ①②③ | 3 |
| Weng YJ 2017(16) | ACU VS LI | 30 min, 5 times per week | WHO 1999 | 29/26 | NM | NM | NM | NM | ①②③ | 2 |
| Guo K 2021(17) | ACE VS LI | q10d | NM | 25/25 | NM | NM | NM | NM | ①②③ | 12 |
| Zhao BT 2020(18) | ACE VS LI | q15d | WHO 1999 | 36/38 | 18/18 | 18/20 | 36.58±6.48 | 37.25±4.26 | ①②③ | 5 |
| Qu FZ 2016(19) | ACE VS LI | qm | CDS 2004 | 31/31 | 8/23 | 7/24 | 47.12±4.22 | 47.17±3.72 | ①②⑥ | 3 |
| Zhang LB 2010(20) | ACE VS LI | qm | CDS 2017 | 56/58 | NM | NM | NM | NM | ①②④⑤⑥ | 6 |
| Nie YL 2016(21) | ACE VS LI | qw | CDS 2010 | 30/30 | 10/20 | 11/19 | 44±13.2 | 43±13.7 | ①② | 3 |
| Wang Y 2021(22) | ACE VS LI | q2w | CDS 2020 | 30/30 | 15/15 | 18/12 | 53.9±8.20 | 51.1±10.0 | ①②③④⑤ | 2 |
| Xue N 2017(23) | ACE VS LI | q15d | CDS 2004 | 43/43 | 18/25 | 20/23 | 52±12 | 53±13 | ①②③④⑤ | 3 |
| Lin LR 2019(24) | ACE VS LI | q4d | CDS 2004 | 30/30 | 18/12 | 16/14 | 49.00±5.10 | 47.67±4.50 | ①②③ | 3 |
| Li YM 2013(25) | ACE VS LI | qm | CDS 2004 | 56/58 | NM | NM | NM | NM | ①②④⑤⑥ | 12 |
| Xu P 2022(26) | BDJ VS LI | 30 min, bid, 5 days per week | WHO 1998 | 20/20 | 10/10 | 12/8 | 53.57±11.21 | 52.34±12.01 | ①②④⑤ | 3 |
| Fang CP 2014(27) | BDJ VS LI | 30 min, bid, 5 days per week | WHO 1999 | 28/30 | NM | NM | NM | NM | ①②③④⑤ | NM |
| Ma XJ 2022(28) | BDJ VS LI | 60 min, qod | NM | 34/32 | 16/18 | 14/18 | 59.18 ± 3.93 | 59.09 ± 5.25 | ①②③④⑤ | 12 |
| Li WH 2019a(29) | BDJ VS LI | 20 min, tiw | WHO 1999 | 49/47 | 15/34 | 14/33 | 56.90±3.94 | 56.34 ± 4.03 | ① | 9 |
| Ren JQ 2014(30) | BDJ VS LI | qod, 5 days per week | ADA 2012 | 8/8 | 1/7 | 2/6 | 63.25±6.86 | 60.38±5.81 | ①②③⑥ | 6 |
| Wu YC 2016(31) | BDJ VS LI | qod, 5 days per week | ADA 2012 | 20/20 | NM | NM | 63.25±6.86 | 60.38 ± 5.81 | ①②③⑥ | 6 |
| Ren QJ 2022(32) | BDJ VS LI | 60 min, qd | NM | 12/13 | NM | NM | 51.67±3.17 | 48.62±4.97 | ①③④⑤⑥ | 3 |
| Wang NN 2021(33) | BDJ VS LI | 20 min, bid | NM | 50/50 | NM | NM | 57.67±5.17 | 57.52±5.29 | ①②④⑤⑥ | NM |
| Jiang F 2023(34) | BDJ VS LI | tiw | NM | 40/40 | NM | NM | NM | NM | ①②⑥ | 12 |
| Shen Y 2020(35) | BDJ VS LI | 30 min, bid, 5 days per week | CDS 2011 | 52/51 | 25/27 | 23/28 | 40.30±2.41 | 40.33±2.39 | ①②④⑤⑥ | 6 |
| Li Q 2022(36) | BDJ VS LI | 30 min, bid, 5 days per week | ADA 2017 | 39/37 | 21/18 | 22/15 | 54.26± 10.81 | 53.67±11.01 | ①②③④⑤⑥ | 6 |
| Shang YH 2017(37) | BDJ VS LI | 60min, five days per week | CDS 2010 | 43/43 | 21/22 | 22/21 | 54 | 55 | ①②④⑤⑥ | 6 |
| Wang WY 2020(38) | BDJ VS LI | 40 min, tiw | NM | 17/15 | 6/11 | 5/10 | 64.52±4.74 | 65.73±4.37 | ①②④⑤⑥ | 3 |
| Liu HY 2019(39) | EAC VS LI | 20 min, 5 times per week | WHO 1999 | 20/20 | 12/8 | 11/9 | 44±4 | 47±6 | ①②③④⑤⑥ | 24 |
| Su W 2019(40) | EAC VS LI | 20 min, tiw | ADA 2018 | 30/30 | 11/19 | 12/18 | 52±2 | 49±2 | ①②④⑤ | 3 |
| Zhai X 2010(41) | EAC VS LI | 20 min, qd | WHO 1999 | 30/30 | 8/22 | 7/23 | NM | NM | ①②③ | 3 |
| Li K 2016(42) | EAC VS LI | 30 min, first 3 days qd, then qod | WHO 1999 | 46/46 | 16/30 | 20/26 | 41±12.2 | 43±11.8 | ①②⑥ | 2 |
| Zhao NN 2015(43) | MSG VS LI | 12 min, tiw | WHO 1999 | 41/42 | 23/18 | 22/20 | 45.65±8.14 | 42.89±6.01 | ②③⑥ | 1 |
| Ji JJ 2018(44) | MSG VS LI | 50 min, tiw | ADA 2014 | 16/18 | NM | NM | 64.06±6.71 | 62.50±6.33 | ①②③④⑤⑥ | 3 |
| Li YL 2016(45) | MSG VS LI | 30 min, qd | WHO 1999 | 30/30 | 16/14 | 18/12 | 47.80±4.32 | 45.60±4.98 | ①② | 3 |
| Hu HJ 2010(46) | MSG VS LI | qod | CDS 2004 | 31/29 | 16/15 | 15/14 | 48.70±4.69 | 51.30±6.95 | ①② | 6 |
| Chen JD 2020(47) | TAJ VS LI | 60 min, qd | NM | 10/10 | 5/5 | 4/6 | 52.40±2.50 | 53.20±4.04 | ①③④⑤⑥ | 4 |
| Luan J 2023(48) | TAJ VS LI | 40-60 min, tiw | CEC-IAPD 2020(49) | 20/20 | 10/10 | 10/10 | 58.41±6.01 | 56.75±5.97 | ①②③④⑤ | 3 |
| Song HN 2013(50) | TAJ VS LI | 50 min, qd | WHO 1999 | 40/40 | NM | NM | NM | NM | ①②③ | 3 |
| Yu ZH 2023(51) | TAJ VS LI | 60 min, tiw | NM | 15/17 | 8/7 | 8/9 | 59.40±5.30 | 59.59±7.22 | ①② | 3 |
| Li WH 2019b (52) | TAJ VS LI | 60 min, 5 times per week | WHO 1999 | 43/44 | 12/31 | 15/29 | 55.72±3.42 | 55.25±3.38 | ① | 6 |
| Note: C: control group, T: treatment group. ACU: acupuncture, ACE: acupoint catgut embedding, BDJ: Baduanjin, LI: lifestyle intervention, EAC: electroacupuncture, MSG: massage, TAJ: Taiji. ①: FPG, ②: 2hPG, ③: HbA1c, ④: TC, ⑤: TG, ⑥: BMI. bid: twice daily; biw: twice weekly; q10d: every 10 days; q15d: every 15 days; q2w: every 2 weeks; q4d: every 4 days; qd: once daily; qm: once monthly; qod: every other day; qw: once weekly; tiw: three times weekly. | | | | | | | | | | |

1. Li Y, Xie K, Zeng X et al. Effect of Zuo's warming Yang acupuncture therapy combined with lifestyle interventions on prediabetes: A randomized controlled trial*.* Complement Ther Med. (2023) 78:102985. doi:10.1016/j.ctim.2023.102985

2. Gong Z, Wang Z, He H. Clinical Observation of Abdominal Acupuncture Treatment in Patients with Prediabetes and Concomitant Overweight/Obesity*.* Inner Mong J Tradit Chin Med. (2024) 53(1):101-103. doi:10.16040/j.cnki.cn15-1101.2024.01.047

3. Li Y, Zheng D, Zuo C. A Clinical Trial of Liji Acupuncture in Patients with Prediabetes*.* J Sichuan Tradit Chin Med. (2020) 38(11):180-181

4. Wang H, Xiao Y, Wei Y et al. The Effect of Balance Acupuncture on the Clinical Outcome of Individuals with Impaired Glucose Tolerance*.* Henan Tradit Chin Med. (2013) 33(12):2202-2203. doi:10.16367/j.issn.1003-5028.2013.12.043

5. Zhu Y, Huang S, Li W et al. Clinical Observation of Impaired Glucose Tolerance with Adult Obesity by using Balance Acupuncture Treatment*.* Prog Mod Biomed. (2016) 16(34):6694-6697. doi:10.13241/j.cnki.pmb.2016.34.024

6. Luo G, Yu Q, Li X. Balance Acupuncture Combined with Life Style Intervention Has Effect on Patients with Impaired Glucose Tolerance*.* New Chin Med. (2019) 51(02):207-209. doi:10.13457/j.cnki.jncm.2019.02.063

7. Zhang H. (2022) Clinical Observation of the Nape Seven Needles Intervention in Prediabetes. [master’s thesis]. [Jinan]: Shangdong University of Traditional Chinese Medicine. doi:10.27282/d.cnki.gsdzu.2022.000770

8. Tan C, Lan W, Lan H et al. Clinical Study on Acupuncture Treatment of People with Phlegm-dampness Constitution and Impaired Glucose Tolerance*.* J Hunan Univ Chin Med. (2019) 39(11):1377-1381. doi:10.3969/j.issn.1674－070X.2019.11.019

9. Ma S. Observation on Therapeutic Effect of Acupuncture Replenishing Spleen Therapy on Prediabetes*.* Reflexol Rehabil Med. (2020) 1(18):46-48

10. Hu Z. (2024) A Controlled Clinical Studyof Acupuncture Interventionin Prediabetes. [master’s thesis]. [Wuhan]: Hubei University of Chinese Medicine. doi:10.27134/d.cnki.ghbzc.2024.000350

11. Sun X, Fang Z. Clinical Observation of Acupuncture Pixu Tangshi Phlegm Dampness and Spleen Deficiency Syndrome in Early Stage of Diabetes Mellitus*.* Clin J Tradit Chin Med. (2018) 30(10):1850-1852. doi:10.16448/j.cjtcm.2018.0567

12. Liu H. Analysis of the effect of acupuncture, laxative stomach and spleen replenishment therapy in pre-diabetes*.* Contemp Med. (2020) 26(10):73-75. doi:10.3969/j.issn.1009-4393.2020.10.027

13. Liu Y. (2017) Clinical study on the treatment of early stage of diabetes with the method of invigorating stomach and invigorating spleen. [master’s thesis]. [Nanjing]: Nanjing University of Chinese Medicine

14. Fan D, Niu Y, Zhang G. Acupuncture for pre-diabetes: a report of 32 cases*.* Tradit Chin Med Res. (2014) 27(6):59-60. doi:10.3969/j.issn.1001-6910.2014.06.30

15. Lan W. (2019) Acupuncture Intervention in Individuals with Impaired Glucose Tolerance and Phlegm-Dampness Constitution: A Clinical Observation. [master’s thesis]. [Wuhan]: Hubei University of Chinese Medicine. doi:10.27138/d.cnki.ghuzc.2019.000325

16. Weng Y, Tang L, He H. Observation on the curative effect of Jin 's three-needle combined with eight methods of linggui in the treatment of patients with impaired glucose regulation*.* China's Naturopathy. (2017) 25(05):16-18. doi:10.19621/j.cnki.11-3555/r.2017.05.012

17. Guo K. Clinical Efficacy and Safety Analysis of Acupoint Catgut Embedding Therapy for Prediabetes*.* Health Guide. (2021) (2):107

18. Zhao B, Zhou A, Chen J, Zhu Y. Observation on the Therapeutic Effect of Taiji Liuhe Acupoint Catgut Embedding in the Treatment of Pre-diabetes*.* J Zhejiang Chin Med Univ. (2020) 44(10):1004-1008. doi:10.16466/j.issn1005-5509.2020.10.017

19. Qu F. (2016) The clinical observation for the catgut implantation atacupoint to treat pre-diabetes. [master’s thesis]. [Zhengzhou]: Henan University of Chinese Medicine

20. Zhang L, Zhang C, Wang S et al. The Effect of Acupoint Catgut Embedding on Sugar Metabolism in the Crowd of Prediabetes*.* J Clin Acupunct Moxibustion. (2010) 26(11):1-4

21. Nie Y. Clinical Study on Acupuncture Embedding Therapy in the Treatment of Obese Patients with Pre-diabetes*.* Asia-Pac Tradit Med. (2016) 12(24):121-122

22. Wang Y. (2021) Observation on the clinical effect of catgut embedding at acupoints in treating pre-diabetes with damp-heat accumulating spleen. [master’s thesis]. [Wuhan]: Hubei University of Chinese Medicine. doi:10.27134/d.cnki.ghbzc.2021.000221

23. Xue N, Zhang J, Xia Z, Da N. Effect and postoperative reactions of acupoint catgut embedding for prediabetes*.* Chin Acupunct Moxibustion. (2017) 37(06):586-590. doi:10.13703/j.0255-2930.2017.06.004

24. Lin L. (2019) Clinical study on acupoint embedding therapy for pre-diabetes. [master’s thesis]. [Guangzhou]: Guangzhou University of Chinese Medicine. doi:10.27044/d.cnki.ggzzu.2019.000061

25. Li Y, Zhang C, Di L et al. Comparative Study on Intervention Sugar Regulation Damaged Crowd Back to Normal Glucose Tolerance*.* Pract Clin J Integr Tradit Chin West Med. (2013) 13(02):3-4+6

26. Xu P. Observation on the Efficacy of Baduanjin Exercise Under the Exercise-Medicine Integration Model in Community Patients with Prediabetes*.* Chin Sci J Database. (2022) (7):0044-0046

27. Fang C, Jiang H, Wang D et al. Effects of eight-sectional exercise in Qigong on prevention of decreased glucose tolerance*.* Tianjin J Tradit Chin Med. (2014) 31(10):588-590

28. Ma X, Li M, Liu L et al. A randomized controlled trial of Baduanjin exercise to reduce the risk of atherosclerotic cardiovascular disease in patients with prediabetes*.* Sci Rep. (2022) 12(1):19338. doi:10.1038/s41598-022-22896-5

29. Li W, Wu Z, Lu L, Pan H. Effects of Ba Duan Jin and Resistance Exercises on Glucose and Insulin Resistance in Patients with Impaired Glucose Regulation*.* World Sci Technol. (2019) 21(06):1251-1256

30. Ren J. (2014) Clinical Research On Method Of Qigong Buduanjin in patients with pre-diabetes. [master’s thesis]. [Nanjing]: Nanjing University of Chinese Medicine

31. Wu Y, Wei Q, Ren J. Clinical Research and Mechanism of Baduanjin on Psychological and Physiological State of Pre-Diabetes*.* Liaoning J Tradit Chin Med. (2016) 43(10):2031-2034. doi:10.13192/j.issn.1000-1719.2016.10.004

32. Ren Q. (2022) Effect of Baduanjin Combined With Resistance Training on Prediabetic. [master’s thesis]. [Shenyang]: Liaoning Normal University. doi:10.27212/d.cnki.glnsu.2022.000429

33. Wang N. Analysis of the Efficacy of Baduanjin Exercise in Prediabetic Individuals and Its Effect on Blood Glucose and Lipid Profiles*.* Chin Sci J Database. (2021) (10):2

34. Jiang F, Sun C, Zheng X, Ding S, Wang R. The Intervention Effect of Baduanjin Exercise Combined with Health Education Based on the Medical Consortium Model on Community-Based Prediabetes*.* Chin Sci J Database: Med Health. (2023) (5):0033-0036

35. Shen Y, Zhang N, Li S. Analysis of the Effect of Baduanjin Applied to Prediabetic Population and Its Impact on Blood Glucose and Lipid Levels*.* Chin J Convalescent Med. (2020) 29(11):1139-1141. doi:10.13517/j.cnki.ccm.2020.11.007

36. Li Q, Wu R, Wang F, Shi B, An R. Clinical observation on intervention effect of Baduanjin on pre-diabetic patients in the community*.* Shanghai J Tradit Chin Med. (2022) 56(05):49-53. doi:10.16305/j.1007-1334.2022.2103082

37. Shang Y, Shang Y, Cai Y, Wu M. A comparative study on application effect of BaDuan Jin and brisk walking in pre-diabetes crowd*.* Nurs Res. (2017) 31(35):4555-4557

38. Wang W. (2020) Effect of Health Qigong Baduanjin Exercise on Energy Metabolism in Individuals with Prediabetes and Impaired Glucose Tolerance: An Observational Study. [master’s thesis]. [Tianjin]: Tianjin University of Sport. doi:10.27364/d.cnki.gttyy.2020.000172

39. Liu H, Hua Y, Li Y, Chen Q. Study of the Effect of Using Electroacupuncture for Patients with Impaired Glucose Tolerance*.* Shanghai J Acupunct Moxibustion. (2019) 38(04):359-363. doi:10.13460/j.issn.1005-0957.2019.04.0359

40. Su W, Shi Y. Therapeutic Observation of Acupuncture at the Ear Center for Early-stage Diabetes*.* Shanghai J Acupunct Moxibustion. (2019) 38(11):1229-1232. doi:10.13460/j.issn.1005-0957.2019.11.1229

41. Zhai X. (2010) Clinical Studies of Electro-acupuncture Intervening People with Impaired Glucose Tolerance. [master’s thesis]. [Beijing]: China Academy of Chinese Medical Sciences

42. Li K, Zhou Z. Observation on the Therapeutic Efficacy of Electroacupuncture in Treating Simple Obesity Complicated by Impaired Glucose Tolerance*.* J Clin Acupunct Moxibustion. (2016) 32(07):40-42. doi:10.19917/j.cnki.1005-0779.2016.07.015

43. Li M, Zhao N. Clinical Value Evaluation of Finger Acupoint Pressing Nursing Combined with Exercise Therapy for Impaired Glucose Tolerance*.* J Clin Nurs. (2015) 14(02):45-47

44. Ji J, Yang S, Lou Q, Liu C. Effect of Traditional Chinese Medicine Meridian Massage on Prediabetes Patients*.* J Hunan Univ Chin Med. (2018) 38(02):160-164

45. Li Y, Huang L, Ni D, Chen W. Effect of Self-Massage on Prediabetes*.* Chin Gen Pract Nurs. (2016) 14(34):3600-3602

46. Hu H, Zhan R, Zhan H. Individualized Basic Nursing Combined with Acupoint Massage for Impaired Glucose Regulation*.* Hebei J Tradit Chin Med. (2010) 32(01):130-131

47. Chen J. (2020) A Study on the Intervention Effect of 24-Pattern ShadowboxingCombined with Resistive Exercise in Patients with pre-diabetes. [master’s thesis]. [Liaoning]: Liaoning Normal University. doi:10.27212/d.cnki.glnsu.2020.000799

48. Luan J. (2023) Study on the intervention effect of Eight-Form Tai Chi on pre-diabetic people underdifferent exercise management modes. [master’s thesis]. [Guangzhou]: Guangzhou Sport University. doi:10.27042/d.cnki.ggztc.2023.000183

49. Chinese Society Of Endocrinology CDS. Intervention for adults with pre-diabetes: a Chinese expert consensus (2023 edition)*.* Chin J Diabetes Mellitus. (2023) 15(6):484-494. doi:10.3760/cma.j.cn115791-20230509-00188

50. Song H. Effect of Tai Chi on Rehabilitation Outcomes in Patients with Impaired Glucose Regulation*.* Chin J Tradit Med Sci Technol. (2013) 20(06):664

51. Yu Z. (2023) Effects of 12 weeks of tai chi exercise oninhibitory control and conversion functionin prediabetic middle-aged and older adults. [master’s thesis]. [Guangzhou]: Guangzhou Sport University. doi:10.27042/d.cnki.ggztc.2023.000251

52. Li W, Wu Z, Jing C, Pan H. Effects of Chen-style taijiquan on cardiorespiratory endurance of pre-diabetic patients*.* China J Tradit Chin Med Pharm. (2019) 34(06):2807-2809

**Appendix** **4. Risk of bias of included studies**

**Supplementary** **Table 3.**

| Study ID | Randomization process | Deviations from intended interventions | Mising outcome data | Measurement of the outcome | Selection of the reported result | Overall Bias |
| --- | --- | --- | --- | --- | --- | --- |
| Li Y 2023 | Low | Some concerns | Low | Low | Low | Some concerns |
| Gong ZF 2024 | Some concerns | Low | Low | Low | Some concerns | Some concerns |
| Li Y 2020 | Some concerns | Low | Low | Low | Some concerns | Some concerns |
| Wang H 2013 | Some concerns | Some concerns | Some concerns | Low | Some concerns | Some concerns |
| Zhu YP 2016 | Some concerns | Low | Some concerns | Low | Some concerns | Some concerns |
| Luo GG 2019 | Some concerns | Some concerns | Low | Low | Some concerns | Some concerns |
| Zhang H 2022 | Low | High | Low | Low | Low | High |
| Tan CJ 2019 | Low | High | High | Low | Some concerns | High |
| Ma SQ 2020 | Some concerns | Low | Low | Low | Some concerns | Some concerns |
| Hu ZB 2024 | Low | Some concerns | Low | Low | Low | Some concerns |
| Sun XJ 2018 | Some concerns | Low | Low | Low | Some concerns | Some concerns |
| Liu H 2020 | Some concerns | Low | Low | Low | Some concerns | Some concerns |
| Liu Y 2017 | Low | High | High | Low | Low | High |
| Fan DS 2014 | Low | Some concerns | Low | Low | Some concerns | Some concerns |
| Lan W 2019 | Low | Some concerns | High | Low | Low | High |
| Weng YJ 2017 | Some concerns | High | Low | Low | Some concerns | High |
| Guo K 2021 | Low | Low | Low | Low | Some concerns | Some concerns |
| Zhao BT 2020 | Low | High | High | High | Some concerns | High |
| Qu FZ 2016 | Low | Some concerns | Some concerns | High | Low | High |
| Zhang LB 2010 | Some concerns | Some concerns | Low | Low | Some concerns | Some concerns |
| Nie YL 2016 | Low | Low | Low | Low | Some concerns | Some concerns |
| Wang Y 2021 | Low | Some concerns | Low | Low | Low | Some concerns |
| Xue N 2017 | Some concerns | Low | Low | Low | Some concerns | Some concerns |
| Lin LR 2019 | Low | Low | Low | Low | Low | Low |
| Li YM 2013 | Some concerns | Some concerns | Low | Low | Some concerns | Some concerns |
| Xu P 2022 | Some concerns | Low | Low | Low | Some concerns | Some concerns |
| Fang CP 2014 | Low | Some concerns | Low | Low | Some concerns | Some concerns |
| Ma XJ 2022 | Low | Some concerns | High | Low | Low | High |
| Li WH 2019a | Low | High | Low | Low | Some concerns | High |
| Ren JQ 2014 | Low | High | Some concerns | Low | Low | High |
| Wu YC 2016 | Some concerns | Low | Low | Low | Some concerns | Some concerns |
| Ren QJ 2022 | Some concerns | Low | Low | Low | Low | Some concerns |
| Wang NN 2021 | Some concerns | Low | Low | Low | Some concerns | Some concerns |
| Jiang F 2023 | Low | Low | Low | Low | Some concerns | Some concerns |
| Shen Y 2020 | Low | Low | Low | Low | Some concerns | Some concerns |
| Li Q 2022 | Low | Some concerns | Low | Low | Some concerns | Some concerns |
| Shang YH 2017 | Low | Low | Low | Low | Some concerns | Some concerns |
| Wang WY 2020 | Some concerns | High | High | Low | Low | High |
| Liu HY 2019 | Some concerns | Low | Low | Low | Some concerns | Some concerns |
| Su W 2019 | Low | Low | Low | Low | Some concerns | Some concerns |
| Zhai X 2010 | Low | Low | Low | Low | Low | Low |
| Li K 2016 | Some concerns | Low | Low | Low | Some concerns | Some concerns |
| Zhao NN 2015 | Some concerns | Low | Low | Low | Some concerns | Some concerns |
| Ji JJ 2018 | Low | High | Low | Low | Some concerns | High |
| Li YL 2016 | Low | Low | Low | Low | Some concerns | Some concerns |
| Hu HJ 2010 | Some concerns | Low | Low | Low | Some concerns | Some concerns |
| Chen JD 2020 | Some concerns | Low | Low | Low | Low | Some concerns |
| Luan J 2023 | Some concerns | High | Low | Low | Low | High |
| Song HN 2013 | Some concerns | Low | Low | Low | Some concerns | Some concerns |
| Yu ZH 2023 | Some concerns | High | High | Low | Low | High |
| Li WH 2019b | Low | Some concerns | Low | Low | Some concerns | Some concerns |

|  | Randomization process | Deviations from intended interventions | Missing outcome data | Measurement of the outcome | Selection of the reported result | Overall Bias |
| --- | --- | --- | --- | --- | --- | --- |
| Assignment to intervention (the 'intention-to-treat' effect) | | | | | | |
| Total number of studies = 51 | | | | | | |
| Low risk | 51% | 51% | 78.4% | 96.1% | 31.4% | 3.9% |
| Some concerns | 49% | 27.5% | 7.8% | 0% | 68.6% | 68.6% |
| High risk | 0% | 21.6% | 13.7% | 3.9% | 0% | 27.5% |

**Appendix 5.** **Evaluation of** **heterogeneity**

| **Supplementary** **Table 4.** Heterogeneity tests for FPG | | | | | |
| --- | --- | --- | --- | --- | --- |
| Treatment | Q | df | P | I² (%) | τ^2^ |
| A | 214.42 | 15 | 0.000 | 93.0 | 0.0753 |
| B | 103.40 | 8 | 0.000 | 92.3 | 0.1558 |
| C | 130.87 | 12 | 0.000 | 90.8 | 0.1312 |
| E | 85.75 | 3 | 0.000 | 96.5 | 0.3032 |
| F | 18.84 | 2 | 0.000 | 89.4 | 0.8180 |
| G | 45.99 | 4 | 0.000 | 91.3 | 0.1360 |
| Overall | 659.24 | 49 | 0.000 | 92.6 | 0.1009 |
| Note A: acupuncture, B: acupoint catgut embedding, C: Baduanjin, D: lifestyle intervention, E: electroacupuncture, F: massage, G: Taiji. | | | | | |

| **Supplementary** **Table 5.** Heterogeneity tests for 2hPG | | | | | |
| --- | --- | --- | --- | --- | --- |
| Treatment | Q | df | P | I² (%) | τ² |
| A | 294.28 | 12 | 0.000 | 95.9 | 0.4598 |
| B | 44.92 | 8 | 0.000 | 82.2 | 0.1289 |
| C | 40.44 | 10 | 0.000 | 75.3 | 0.2769 |
| E | 16.46 | 3 | 0.001 | 81.8 | 0.2176 |
| F | 3.47 | 3 | 0.325 | 13.6 | 0.0500 |
| G | 0.14 | 2 | 0.935 | 0.0 | 0.0000 |
| **Overall** | **482.21** | **43** | **0.000** | **91.1** | **0.3457** |
| Note A: acupuncture, B: acupoint catgut embedding, C: Baduanjin, D: lifestyle intervention, E: electroacupuncture, F: massage, G: Taiji. | | | | | |

| **Supplementary** **Table 6.** Heterogeneity tests for HbA1c | | | | | |
| --- | --- | --- | --- | --- | --- |
| Treatment | Q | df | P | I² (%) | τ² |
| A | 158.26 | 7 | 0.000 | 95.6 | 0.1602 |
| B | 25.40 | 4 | 0.000 | 84.3 | 0.1087 |
| C | 44.78 | 5 | 0.000 | 88.8 | 0.0488 |
| E | 1.66 | 1 | 0.198 | 39.8 | 0.0156 |
| F | 0.02 | 1 | 0.888 | 0.0 | 0.0000 |
| G | 2.34 | 2 | 0.310 | 14.6 | 0.0078 |
| Overall | 281.06 | 25 | 0.000 | 91.1 | 0.0946 |
| Note A: acupuncture, B: acupoint catgut embedding, C: Baduanjin, D: lifestyle intervention, E: electroacupuncture, F: massage, G: Taiji. | | | | | |

| **Supplementary** **Table 7.** Heterogeneity tests for BMI | | | | | |
| --- | --- | --- | --- | --- | --- |
| Treatment | Q | df | P | I² (%) | τ² |
| A | 42.62 | 3 | 0.000 | 93.0 | 2.8921 |
| B | 2.40 | 2 | 0.301 | 16.8 | 0.0183 |
| C | 53.64 | 8 | 0.000 | 85.1 | 0.4296 |
| E | 0.18 | 1 | 0.672 | 0.0 | 0.0000 |
| F | 20.62 | 1 | 0.000 | 95.1 | 20.8494 |
| G | 0.00 | 0 | - | - | 0.0000 |
| Overall | 375.14 | 20 | 0.000 | 94.7 | 1.7018 |
| Note A: acupuncture, B: acupoint catgut embedding, C: Baduanjin, D: lifestyle intervention, E: electroacupuncture, F: massage, G: Taiji. | | | | | |

| **Supplementary** **Table 8.** Heterogeneity tests for TC | | | | | |
| --- | --- | --- | --- | --- | --- |
| Treatment | Q | df | P | I² (%) | τ² |
| A | 8.59 | 5 | 0.127 | 41.8 | 0.0147 |
| B | 19.94 | 3 | 0.000 | 85.0 | 0.1485 |
| C | 43.60 | 8 | 0.000 | 81.6 | 0.1173 |
| E | 15.73 | 1 | 0.000 | 93.6 | 0.3072 |
| F | 0.00 | 0 | - | - | 0.0000 |
| G | 0.40 | 1 | 0.529 | 0.0 | 0.0000 |
| Overall | 92.84 | 23 | 0.000 | 75.2 | 0.0680 |
| Note A: acupuncture, B: acupoint catgut embedding, C: Baduanjin, D: lifestyle intervention, E: electroacupuncture, F: massage, G: Taiji. | | | | | |

| **Supplementary** **Table 9.** Heterogeneity tests for TG | | | | | |
| --- | --- | --- | --- | --- | --- |
| Treatment | Q | df | P | I² (%) | τ² |
| A | 14.12 | 4 | 0.007 | 71.7 | 0.0242 |
| B | 48.39 | 3 | 0.000 | 93.8 | 0.1844 |
| C | 118.55 | 8 | 0.000 | 93.3 | 0.1923 |
| E | 12.37 | 1 | 0.000 | 91.9 | 0.1942 |
| F | 0.00 | 0 | - | - | 0.0000 |
| G | 0.01 | 1 | 0.904 | 0.0 | 0.0000 |
| Overall | 210.69 | 22 | 0.000 | 89.6 | 0.0939 |
| Note A: acupuncture, B: acupoint catgut embedding, C: Baduanjin, D: lifestyle intervention, E: electroacupuncture, F: massage, G: Taiji. | | | | | |

| **Supplementary** **Table 10.** Meta-regression analysis for FPG | | | | | |  |
| --- | --- | --- | --- | --- | --- | --- |
| Variable | Coefficient | SE | z | P | 95%CI | |
| Frequency | -0.1146 | 0.1318 | -0.87 | 0.385 | (-0.3730, 0.1438) | |
| Duration | -0.1021 | 0.1233 | -0.83 | 0.408 | (-0.3439, 0.1396) | |
| Sample size | 0.1880 | 0.2057 | 0.91 | 0.361 | (-0.2151, 0.5911) | |
| Age | 0.0837 | 0.1241 | 0.67 | 0.500 | (-0.1596, 0.3269) | |
| Constant | -0.5119 | 0.3588 | -1.43 | 0.154 | (-1.2151, 0.1913) | |

**Appendix 6. Meta-regression analysis**

| **Supplementary** **Table 11.** Meta-regression analysis for 2hPG | | | | | |
| --- | --- | --- | --- | --- | --- |
| Variable | Coefficient | SE | z | P | 95% CI |
| Frequency | -0.3258 | 0.1742 | -1.87 | 0.061 | (-0.6672, 0.0156) |
| Duration | -0.0249 | 0.1732 | -0.14 | 0.886 | (-0.3643, 0.3146) |
| Sample size | 0.6309 | 0.2432 | 2.59 | **0.009** | (0.1542, 1.1077) |
| Age | 0.1468 | 0.1591 | 0.92 | 0.356 | (-0.1649, 0.4586) |
| Constant | -1.1396 | 0.4146 | -2.75 | 0.006 | (-1.9522, -0.3270) |

| **Supplementary** **Table 12.** Meta-regression analysis for HbA1c | | | | | |
| --- | --- | --- | --- | --- | --- |
| Variable | Coefficient | SE | z | P | 95% CI |
| Frequency | 0.1762 | 0.1087 | 1.62 | 0.105 | (-0.0367, 0.3892) |
| Duration | -0.0400 | 0.0948 | -0.42 | 0.673 | (-0.2258, 0.1459) |
| Sample size | dropped |  |  |  |  |
| Age | -0.0734 | 0.1047 | -0.70 | 0.483 | (-0.2787, 0.1319) |
| Constant | -0.6087 | 0.2314 | -2.63 | 0.009 | (-1.0622, -0.1551) |

| **Supplementary** **Table 13.** Meta-regression analysis for BMI | | | | | |
| --- | --- | --- | --- | --- | --- |
| Variable | Coefficient | SE | z | P | 95% CI |
| Frequency | 0.4068 | 1.3517 | 0.30 | 0.763 | (-2.2425, 3.0561) |
| Duration | -0.3098 | 1.0884 | -0.28 | 0.776 | (-2.4430, 1.8233) |
| Sample size | 0.1599 | 0.9118 | 0.18 | 0.861 | (-1.6271, 1.9469) |
| Age | 1.7407 | 0.7221 | 2.41 | **0.016** | (0.3253, 3.1560) |
| Constant | -5.9187 | 2.4141 | -2.45 | 0.014 | (-10.6504, -1.1871) |

| **Supplementary** **Table 14.** Meta-regression analysis for TC | | | | | |
| --- | --- | --- | --- | --- | --- |
| Variable | Coefficient | SE | z | P | 95% CI |
| Frequency | 0.2020 | 0.1433 | 1.41 | 0.159 | (-0.0789, 0.4828) |
| Duration | -0.1274 | 0.1249 | -1.02 | 0.308 | (-0.3723, 0.1174) |
| Sample size | 0.0414 | 0.1490 | 0.28 | 0.781 | (-0.2507, 0.3334) |
| Age | 0.1644 | 0.1781 | 0.92 | 0.356 | (-0.1846, 0.5134) |
| Constant | -0.9666 | 0.5643 | -1.71 | 0.087 | (-2.0726, 0.1394) |

| **Supplementary** **Table 15.** Meta-regression analysis for TG | | | | | |
| --- | --- | --- | --- | --- | --- |
| Variable | Coefficient | SE | z | P | 95% CI |
| Frequency | 0.1598 | 0.1573 | 1.02 | 0.310 | (-0.1484, 0.4681) |
| Duration | -0.1207 | 0.1397 | -0.86 | 0.387 | (-0.3945, 0.1530) |
| Sample size | -0.0402 | 0.1825 | -0.22 | 0.826 | (-0.3978, 0.3174) |
| Age | -0.1924 | 0.1876 | -1.03 | 0.305 | (-0.5601, 0.1752) |
| Constant | -0.1107 | 0.5888 | -0.19 | 0.851 | (-1.2647, 1.0432) |

**Appendix 7.** **League tables of outcomes**

| **Supplementary** **Table 16.** The league table of FPG | | | | | | |
| --- | --- | --- | --- | --- | --- | --- |
| F | 0.77 (-0.04,1.58) | 0.99 (0.28,1.70) | 1.04 (0.35,1.74) | 1.10 (0.29,1.90) | 1.24 (0.51,1.96) | 1.49 (0.84,2.14) |
| -0.77 (-1.58,0.04) | E | 0.22 (-0.33,0.78) | 0.27 (-0.26,0.81) | 0.33 (-0.35,1.00) | 0.47 (-0.11,1.05) | 0.72 (0.24,1.20) |
| -0.99 (-1.70,-0.28) | -0.22 (-0.78,0.33) | C | 0.05 (-0.31,0.42) | 0.10 (-0.45,0.66) | 0.25 (-0.18,0.67) | 0.50 (0.22,0.78) |
| -1.04 (-1.74,-0.35) | -0.27 (-0.81,0.26) | -0.05 (-0.42,0.31) | A | 0.05 (-0.48,0.59) | 0.19 (-0.21,0.60) | 0.45 (0.20,0.69) |
| -1.10 (-1.90,-0.29) | -0.33 (-1.00,0.35) | -0.10 (-0.66,0.45) | -0.05 (-0.59,0.48) | G | 0.14 (-0.44,0.72) | 0.39 (-0.08,0.87) |
| -1.24 (-1.96,-0.51) | -0.47 (-1.05,0.11) | -0.25 (-0.67,0.18) | -0.19 (-0.60,0.21) | -0.14 (-0.72,0.44) | B | 0.25 (-0.07,0.58) |
| -1.49 (-2.14,-0.84) | -0.72 (-1.20,-0.24) | -0.50 (-0.78,-0.22) | -0.45 (-0.69,-0.20) | -0.39 (-0.87,0.08) | -0.25 (-0.58,0.07) | D |
| Note A: acupuncture, B: acupoint catgut embedding, C: Baduanjin, D: lifestyle intervention, E: electroacupuncture, F: massage, G: Taiji. | | | | | | |

| **Supplementary** **Table 17.** The league table of 2hPG | | | | | | |
| --- | --- | --- | --- | --- | --- | --- |
| F | 0.43 (-0.63,1.48) | 0.84 (-0.40,2.08) | 0.92 (0.01,1.84) | 0.94 (-0.01,1.88) | 1.27 (0.33,2.20) | 1.80 (0.96,2.63) |
| -0.43 (-1.48,0.63) | E | 0.42 (-0.70,1.53) | 0.50 (-0.24,1.24) | 0.51 (-0.26,1.28) | 0.84 (0.07,1.61) | 1.37 (0.72,2.02) |
| -0.84 (-2.08,0.40) | -0.42 (-1.53,0.70) | G | 0.08 (-0.89,1.06) | 0.09 (-0.91,1.10) | 0.43 (-0.58,1.43) | 0.95 (0.04,1.87) |
| -0.92 (-1.84,-0.01) | -0.50 (-1.24,0.24) | -0.08 (-1.06,0.89) | A | 0.01 (-0.55,0.57) | 0.34 (-0.21,0.89) | 0.87 (0.51,1.23) |
| -0.94 (-1.88,0.01) | -0.51 (-1.28,0.26) | -0.09 (-1.10,0.91) | -0.01 (-0.57,0.55) | C | 0.33 (-0.27,0.93) | 0.86 (0.43,1.29) |
| -1.27 (-2.20,-0.33) | -0.84 (-1.61,-0.07) | -0.43 (-1.43,0.58) | -0.34 (-0.89,0.21) | -0.33 (-0.93,0.27) | B | 0.53 (0.11,0.95) |
| -1.80 (-2.63,-0.96) | -1.37 (-2.02,-0.72) | -0.95 (-1.87,-0.04) | -0.87 (-1.23,-0.51) | -0.86 (-1.29,-0.43) | -0.53 (-0.95,-0.11) | D |
| Note A: acupuncture, B: acupoint catgut embedding, C: Baduanjin, D: lifestyle intervention, E: electroacupuncture, F: massage, G: Taiji. | | | | | | |

| **Supplementary** **Table 18.** The league table of HbA1c | | | | | | |
| --- | --- | --- | --- | --- | --- | --- |
| A | 0.19 (-0.60,0.97) | 0.34 (-0.61,1.30) | 0.37 (-0.87,1.61) | 0.43 (-0.64,1.50) | 0.59 (-0.14,1.32) | 0.80 (0.32,1.27) |
| -0.19 (-0.97,0.60) | B | 0.15 (-0.88,1.19) | 0.18 (-1.12,1.48) | 0.24 (-0.90,1.38) | 0.40 (-0.43,1.24) | 0.61 (-0.02,1.23) |
| -0.34 (-1.30,0.61) | -0.15 (-1.19,0.88) | G | 0.03 (-1.38,1.44) | 0.09 (-1.18,1.35) | 0.25 (-0.74,1.25) | 0.45 (-0.37,1.28) |
| -0.37 (-1.61,0.87) | -0.18 (-1.48,1.12) | -0.03 (-1.44,1.38) | F | 0.06 (-1.43,1.55) | 0.22 (-1.05,1.49) | 0.42 (-0.72,1.57) |
| -0.43 (-1.50,0.64) | -0.24 (-1.38,0.90) | -0.09 (-1.35,1.18) | -0.06 (-1.55,1.43) | E | 0.17 (-0.94,1.27) | 0.37 (-0.59,1.33) |
| -0.59 (-1.32,0.14) | -0.40 (-1.24,0.43) | -0.25 (-1.25,0.74) | -0.22 (-1.49,1.05) | -0.17 (-1.27,0.94) | C | 0.20 (-0.35,0.75) |
| -0.80 (-1.27,-0.32) | -0.61 (-1.23,0.02) | -0.45 (-1.28,0.37) | -0.42 (-1.57,0.72) | -0.37 (-1.33,0.59) | -0.20 (-0.75,0.35) | D |
| Note A: acupuncture, B: acupoint catgut embedding, C: Baduanjin, D: lifestyle intervention, E: electroacupuncture, F: massage, G: Taiji. | | | | | | |

| **Supplementary** **Table 19.** The league table of BMI | | | | | | |
| --- | --- | --- | --- | --- | --- | --- |
| E | 2.51 (-0.72,5.73) | 3.38 (0.74,6.02) | 5.04 (2.64,7.44) | 5.42 (2.66,8.18) | 5.76 (2.03,9.48) | 5.82 (3.63,8.00) |
| -2.51 (-5.73,0.72) | F | 0.87 (-1.92,3.66) | 2.54 (-0.03,5.10) | 2.92 (-0.01,5.84) | 3.25 (-0.58,7.09) | 3.31 (0.95,5.68) |
| -3.38 (-6.02,-0.74) | -0.87 (-3.66,1.92) | A | 1.67 (-0.11,3.44) | 2.04 (-0.20,4.28) | 2.38 (-0.98,5.74) | 2.44 (0.96,3.92) |
| -5.04 (-7.44,-2.64) | -2.54 (-5.10,0.03) | -1.67 (-3.44,0.11) | C | 0.38 (-1.57,2.33) | 0.72 (-2.46,3.89) | 0.78 (-0.21,1.76) |
| -5.42 (-8.18,-2.66) | -2.92 (-5.84,0.01) | -2.04 (-4.28,0.20) | -0.38 (-2.33,1.57) | B | 0.34 (-3.12,3.79) | 0.40 (-1.29,2.08) |
| -5.76 (-9.48,-2.03) | -3.25 (-7.09,0.58) | -2.38 (-5.74,0.98) | -0.72 (-3.89,2.46) | -0.34 (-3.79,3.12) | G | 0.06 (-2.96,3.08) |
| -5.82 (-8.00,-3.63) | -3.31 (-5.68,-0.95) | -2.44 (-3.92,-0.96) | -0.78 (-1.76,0.21) | -0.40 (-2.08,1.29) | -0.06 (-3.08,2.96) | D |
| Note A: acupuncture, B: acupoint catgut embedding, C: Baduanjin, D: lifestyle intervention, E: electroacupuncture, F: massage, G: Taiji. | | | | | | |

| **Supplementary** **Table 21.** The league table of TG | | | | | | |
| --- | --- | --- | --- | --- | --- | --- |
| B | 0.10 (-0.37,0.56) | 0.07 (-0.97,1.11) | 0.15 (-0.54,0.83) | 0.25 (-0.42,0.92) | 0.31 (-0.21,0.84) | 0.41 (0.03,0.80) |
| -0.10 (-0.56,0.37) | C | -0.03 (-1.02,0.97) | 0.05 (-0.58,0.68) | 0.15 (-0.46,0.76) | 0.21 (-0.23,0.66) | 0.31 (0.05,0.57) |
| -0.07 (-1.11,0.97) | 0.03 (-0.97,1.02) | F | 0.07 (-1.05,1.19) | 0.18 (-0.93,1.29) | 0.24 (-0.79,1.27) | 0.34 (-0.62,1.30) |
| -0.15 (-0.83,0.54) | -0.05 (-0.68,0.58) | -0.07 (-1.19,1.05) | G | 0.10 (-0.69,0.89) | 0.17 (-0.51,0.84) | 0.27 (-0.30,0.84) |
| -0.25 (-0.92,0.42) | -0.15 (-0.76,0.46) | -0.18 (-1.29,0.93) | -0.10 (-0.89,0.69) | E | 0.06 (-0.59,0.72) | 0.16 (-0.39,0.71) |
| -0.31 (-0.84,0.21) | -0.21 (-0.66,0.23) | -0.24 (-1.27,0.79) | -0.17 (-0.84,0.51) | -0.06 (-0.72,0.59) | A | 0.10 (-0.26,0.46) |
| -0.41 (-0.80,-0.03) | -0.31 (-0.57,-0.05) | -0.34 (-1.30,0.62) | -0.27 (-0.84,0.30) | -0.16 (-0.71,0.39) | -0.10 (-0.46,0.26) | D |
| Note A: acupuncture, B: acupoint catgut embedding, C: Baduanjin, D: lifestyle intervention, E: electroacupuncture, F: massage, G: Taiji. | | | | | | |

| **Supplementary** **Table 20.** The league table of TC | | | | | | |
| --- | --- | --- | --- | --- | --- | --- |
| E | 0.17 (-0.49,0.82) | 0.24 (-0.35,0.83) | 0.39 (-0.78,1.56) | 0.35 (-0.26,0.96) | 0.25 (-0.54,1.04) | 0.54 (0.01,1.07) |
| -0.17 (-0.82,0.49) | B | 0.07 (-0.40,0.55) | 0.22 (-0.89,1.34) | 0.18 (-0.32,0.68) | 0.08 (-0.62,0.79) | 0.37 (-0.02,0.77) |
| -0.24 (-0.83,0.35) | -0.07 (-0.55,0.40) | C | 0.15 (-0.93,1.23) | 0.11 (-0.30,0.52) | 0.01 (-0.63,0.65) | 0.30 (0.04,0.57) |
| -0.39 (-1.56,0.78) | -0.22 (-1.34,0.89) | -0.15 (-1.23,0.93) | F | -0.04 (-1.13,1.05) | -0.14 (-1.34,1.06) | 0.15 (-0.90,1.20) |
| -0.35 (-0.96,0.26) | -0.18 (-0.68,0.32) | -0.11 (-0.52,0.30) | 0.04 (-1.05,1.13) | A | -0.10 (-0.76,0.57) | 0.19 (-0.12,0.51) |
| -0.25 (-1.04,0.54) | -0.08 (-0.79,0.62) | -0.01 (-0.65,0.63) | 0.14 (-1.06,1.34) | 0.10 (-0.57,0.76) | G | 0.29 (-0.30,0.88) |
| -0.54 (-1.07,-0.01) | -0.37 (-0.77,0.02) | -0.30 (-0.57,-0.04) | -0.15 (-1.20,0.90) | -0.19 (-0.51,0.12) | -0.29 (-0.88,0.30) | D |
| Note A: acupuncture, B: acupoint catgut embedding, C: Baduanjin, D: lifestyle intervention, E: electroacupuncture, F: massage, G: Taiji. | | | | | | |

**Appendix 8. Results ranking**

 **Supplementary Figure 9.** The cumulative probability ranking and SUCRA figure for FPG. Note A: acupuncture, B: acupoint catgut embedding, C: Baduanjin, D: lifestyle intervention, E: electroacupuncture, F: massage, G: Taiji.

| **Supplementary** **Table 22.** The cumulative probability ranking and SUCRA table for FPG | | | |
| --- | --- | --- | --- |
| Treatment | SUCRA (%) | PrBest (%) | MeanRank |
| F | 99.3 | 96.6 | 1.0 |
| E | 73.7 | 3.0 | 2.6 |
| C | 55.8 | 0.1 | 3.7 |
| A | 49.3 | 0.0 | 4.0 |
| G | 43.1 | 0.2 | 4.4 |
| B | 26.9 | 0.0 | 5.4 |
| D | 2.0 | 0.0 | 6.9 |
| Note A: acupuncture, B: acupoint catgut embedding, C: Baduanjin, D: lifestyle intervention, E: electroacupuncture, F: massage, G: Taiji. | | | |

**Supplementary Figure 10.** The cumulative probability ranking and SUCRA figure for 2hPG. Note A: acupuncture, B: acupoint catgut embedding, C: Baduanjin, D: lifestyle intervention, E: electroacupuncture, F: massage, G: Taiji.

| **Supplementary** **Table 23.** The cumulative probability ranking and SUCRA table for 2hPG | | | |
| --- | --- | --- | --- |
| Treatment | SUCRA (%) | PrBest (%) | MeanRank |
| F | 94.1 | 74.7 | 1.4 |
| E | 79.5 | 18.8 | 2.2 |
| G | 54.1 | 5.8 | 3.8 |
| A | 49.3 | 0.3 | 4.0 |
| C | 48.2 | 0.3 | 4.1 |
| B | 24.2 | 0.0 | 5.5 |
| D | 0.4 | 0.0 | 7.0 |
| Note A: acupuncture, B: acupoint catgut embedding, C: Baduanjin, D: lifestyle intervention, E: electroacupuncture, F: massage, G: Taiji. | | | |

 **Supplementary Figure 11.**The cumulative probability ranking and SUCRA figure for HbA1c. Note A: acupuncture, B: acupoint catgut embedding, C: Baduanjin, D: lifestyle intervention, E: electroacupuncture, F: massage, G: Taiji.

| **Supplementary** **Table 24.** The cumulative probability ranking and SUCRA table for HbA1c | | | |
| --- | --- | --- | --- |
| Treatment | SUCRA (%) | PrBest (%) | MeanRank |
| A | 81.8 | 37.1 | 2.1 |
| B | 66.4 | 17.3 | 3.0 |
| G | 54.2 | 13.3 | 3.7 |
| F | 51.8 | 18.9 | 3.9 |
| E | 47.3 | 12.5 | 4.2 |
| C | 34.0 | 0.9 | 5.0 |
| D | 14.4 | 0.0 | 6.1 |
| Note A: acupuncture, B: acupoint catgut embedding, C: Baduanjin, D: lifestyle intervention, E: electroacupuncture, F: massage, G: Taiji. | | | |

 **Supplementary Figure 12.**The cumulative probability ranking and SUCRA figure for BMI. Note A: acupuncture, B: acupoint catgut embedding, C: Baduanjin, D: lifestyle intervention, E: electroacupuncture, F: massage, G: Taiji.

| **Supplementary** **Table 25.** The cumulative probability ranking and SUCRA table for BMI | | | |
| --- | --- | --- | --- |
| Treatment | SUCRA (%) | PrBest (%) | MeanRank |
| E | 98.8 | 92.9 | 1.1 |
| F | 78.4 | 6.7 | 2.3 |
| A | 68.7 | 0.3 | 2.9 |
| C | 38.6 | 0.0 | 4.7 |
| B | 27.7 | 0.0 | 5.3 |
| G | 23.3 | 0.1 | 5.6 |
| D | 14.6 | 0.0 | 6.1 |
| Note A: acupuncture, B: acupoint catgut embedding, C: Baduanjin, D: lifestyle intervention, E: electroacupuncture, F: massage, G: Taiji. | | | |

**Supplementary Figure 13.**
The cumulative probability ranking and SUCRA figure for TC. Note A: acupuncture, B: acupoint catgut embedding, C: Baduanjin, D: lifestyle intervention, E: electroacupuncture, F: massage, G: Taiji.

| **Supplementary** **Table 26.** The cumulative probability ranking and SUCRA table for TC | | | |
| --- | --- | --- | --- |
| Treatment | SUCRA (%) | PrBest (%) | MeanRank |
| E | 79.7 | 44.2 | 2.2 |
| B | 65.1 | 15.9 | 3.1 |
| C | 56.8 | 4.5 | 3.6 |
| G | 53.4 | 15.0 | 3.8 |
| A | 41.4 | 1.9 | 4.5 |
| F | 41.4 | 18.6 | 4.5 |
| D | 12.2 | 0.0 | 6.3 |
| Note A: acupuncture, B: acupoint catgut embedding, C: Baduanjin, D: lifestyle intervention, E: electroacupuncture, F: massage, G: Taiji. | | | |

**Supplementary Figure 14.**
The cumulative probability ranking and SUCRA figure for TG. Note A: acupuncture, B: acupoint catgut embedding, C: Baduanjin, D: lifestyle intervention, E: electroacupuncture, F: massage, G: Taiji.

| **Supplementary** **Table 27.** The cumulative probability ranking and SUCRA table for TG | | | |
| --- | --- | --- | --- |
| Treatment | SUCRA (%) | PrBest (%) | MeanRank |
| B | 74.9 | 28.1 | 2.5 |
| C | 64.9 | 9.7 | 3.1 |
| F | 59.8 | 35.0 | 3.4 |
| G | 55.6 | 16.6 | 3.7 |
| E | 43.3 | 9.0 | 4.4 |
| A | 34.6 | 1.7 | 4.9 |
| D | 17.0 | 0.0 | 6.0 |
| Note A: acupuncture, B: acupoint catgut embedding, C: Baduanjin, D: lifestyle intervention, E: electroacupuncture, F: massage, G: Taiji. | | | |

**Appendix 9. Funnel plot of treatment effects for all outcomes**

**Supplementary Figure 15.** The funnel plots of treatment effects for all outcomes. a: FPG, b: 2hPG, c: HbA1c, d: BMI, e: TC, f: TG. A: acupuncture, B: acupoint catgut embedding, C: Baduanjin, D: lifestyle intervention, E: electroacupuncture, F: massage, G: Taiji.
